# Recent Advances in Nanoparticle Development for Drug Delivery: A Comprehensive Review of Polycaprolactone-Based Multi-Arm Architectures

**DOI:** 10.3390/polym15081835

**Published:** 2023-04-10

**Authors:** Ridouan El Yousfi, Mohamed Brahmi, Mohammed Dalli, Nafea Achalhi, Omar Azougagh, Abdesselam Tahani, Rachid Touzani, Abderrahmane El Idrissi

**Affiliations:** 1 Laboratory Applied Chemistry and Environmental (LCAE-URAC18), Faculty of Sciences of Oujda, University Mohamed Premier, Oujda 60000, Morocco; 2 Physical Chemistry of Natural Substances and Process Team, Laboratory of Applied Chemistry and Environment (LCAE-CPSUNAP), Department of Chemistry, Faculty of Sciences, University Mohamed Premier, Oujda 60000, Morocco; 3 Laboratory of Microbiology, Faculty of Medicine and Pharmacy, University Mohamed Premier, Oujda 60000, Morocco; 4 Laboratory of Molecular Chemistry, Materials and Environment (LMCME), Department of Chemistry, Faculty Multidisciplinary Nador, University Mohamed Premier, P. B. 300, Nador 62700, Morocco

**Keywords:** self-assembly, drug delivery, vesicles, multi-arm polymers, polycaprolactone

## Abstract

Controlled drug delivery is a crucial area of study for improving the targeted availability of drugs; several polymer systems have been applied for the formulation of drug delivery vehicles, including linear amphiphilic block copolymers, but with some limitations manifested in their ability to form only nanoaggregates such as polymersomes or vesicles within a narrow range of hydrophobic/hydrophilic balance, which can be problematic. For this, multi-arm architecture has emerged as an efficient alternative that overcame these challenges, with many interesting advantages such as reducing critical micellar concentrations, producing smaller particles, allowing for various functional compositions, and ensuring prolonged and continuous drug release. This review focuses on examining the key variables that influence the customization of multi-arm architecture assemblies based on polycaprolactone and their impact on drug loading and delivery. Specifically, this study focuses on the investigation of the structure–property relationships in these formulations, including the thermal properties presented by this architecture. Furthermore, this work will emphasize the importance of the type of architecture, chain topology, self-assembly parameters, and comparison between multi-arm structures and linear counterparts in relation to their impact on their performance as nanocarriers. By understanding these relationships, more effective multi-arm polymers can be designed with appropriate characteristics for their intended applications.

## 1. Introduction

The technology for the formation of nanoaggregates aimed at nanoencapsulation and drug delivery is an area that has garnered significant interest as it involves highly sensitive application domains [1,2,3]. Nanoparticles are submicron-sized structures that allow for the controlled release of drugs within the body, thus offering protection against non-specific interactions and enzymatic degradation. These aggregates can be manufactured from a variety of materials such as polymers, lipids, metals, and ceramics, thereby offering a wide range of structures and properties [4,5].

The complexity of certain diseases, as well as the inherent toxicity of certain drugs, has led to a growing interest in the development and optimization of drug delivery systems. Polymeric nanoparticles are a key tool to improve the bioavailability of drugs or produce site-specific effects [6].

Obviously, the development of these nanoaggregates aimed to improve the efficiency of drug encapsulation and facilitate the methods and conditions of drug administration. This has driven scientists to develop new materials, with the most well-known nanocarriers being linear block amphiphilic copolymers. These were first used in the 1980s for the formation of micelles in solution [7]. This type of nanoparticle allowed for the improvement of the solubility of lipophilic drugs that are poorly soluble in water, as well as an increase in their efficacy and bioavailability [8]. Linear polymers have also been able to form other types of smart nanocarriers that are responsive to stimuli, carrying chemical entities sensitive to various conditions such as light, pH, temperature, etc [9]. Linear polymers, despite their advantages, have always been limited by their low encapsulation capacity and high micellar concentration; furthermore, the resulting micelles are only able to encapsulate one type of drug, whether it is hydrophobic or hydrophilic [10].

These limitations have prompted researchers to explore new strategies to enhance the efficiency of drug encapsulation and optimize the performance of drug delivery systems based on amphiphilic copolymers. Several approaches have been developed recently, such as the use of smart linear polymers, modification of micelle structures, or the combination of multiple types of polymers to improve drug encapsulation capacity. These advances have opened up new perspectives in the field of drug delivery, offering new options for the treatment of various diseases [11,12,13,14].

Scientific research has further evolved towards designing new non-linear architectures of copolymers [15], including star [16], comb [17,18], brush [19,20], and hyperbranched structures such as dendrimers [21,22]. This diversity in architecture has overcome certain limitations in drug delivery by personalizing nanoparticles with greater precision. These structures have unique physical and chemical properties that make them attractive for use in drug delivery systems [23]. Dendrimers, for instance, possess a highly branched, tree-like structure that provides precise control over their size and surface properties [24]. They can be functionalized with a variety of molecules, making them ideal for targeted drug delivery. Similarly, star, comb, and brush copolymers have unique structural features that make these types of polymers suitable for drug delivery applications. These architectures have a high degree of branching, which provides a large number of functional groups on their surface. This enables the copolymers to encapsulate and deliver drugs with greater efficiency and to target specific tissues or cells in the body [25]. In addition to offering unique physical and chemical properties, multi-arm copolymers have advantages over traditional linear copolymers in terms of mechanical stability and controlled drug release [26]. The high degree of branching in these structures provides mechanical stability [27,28], which allows them to withstand the harsh conditions of the body’s environment. Moreover, the tunable surface chemistry of these structures allows for the controlled release of drugs over a specified period, which is essential for effective drug therapy [29,30].

Polymer science has played an important role in this advancement, enabling a better understanding of the pharmacokinetic behavior of branched architecture-based nanoparticles and their progression in in vitro and in vivo evaluations [31,32,33]. 

Among the various materials used for the fabrication of nanoparticles, polycaprolactone (PCL) has gained considerable attention due to its biodegradability [34,35], biocompatibility [34,36,37], and ease of synthesis [38,39]. Multi-arm architectures based on PCL have been explored in recent years as a strategy to enhance the drug delivery efficiency of nanoparticles [40,41]. The multi-arm architecture offers several advantages over linear and dendritic polymers, such as increased stability, improved drug loading, and enhanced cellular uptake [42]. Several studies have reported the successful use of multi-arm PCL-based nanoparticles for the delivery of various drugs, including anticancer agents, antibiotics, and anti-inflammatory agents [32,43].

**Figure 1:**
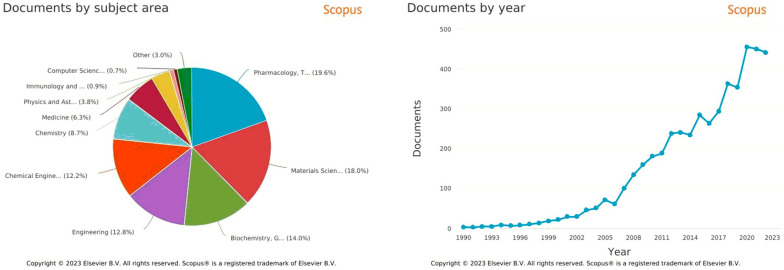
Bibliographic metrics from Scopus (https://www.scopus.com/, accessed on 26 February 2023), with “polycaprolactone multi arms for drug delivery” keywords, from 1990 to 2022.

Significant progress has been made in understanding the nanocarrier–architecture relationship [44,45,46], which can facilitate the development of stable formulations with enhanced drug bioavailability and safety. The variation of characteristics depending on architecture contributes to a growing interest of the scientific community towards these macromolecules. An analysis of the literature shows that the field has undergone a major expansion from a small group to a growing list of researchers, as reflected by the number of publications in recent years (Figure 1).

This comprehensive review aims to provide an in-depth analysis of the current advancements and opportunities in the development of PCL-based multi-arm architectures for drug delivery, with the hope that formulations based on branched architectures will be studied more in depth. We will discuss the various methods of synthesis, properties, and performance of PCL-based multi-arm nanoparticles in drug delivery. Through this review, we hope to provide a better understanding of the potential of PCL-based multi-arm architectures as a versatile drug delivery platform and to inspire further research and development in this area.

## 2. Block Copolymer Self-Assembly: Strategies for Controlling Nanostructure Formation

The self-assembly of copolymers, whether linear or multi-arm, is a spontaneous process in which molecular units transform spontaneously into ordered structures through local interactions [47,48]. This process is well-known biologically in living cells, where it occurs during the formation of the DNA double helix, the folding of polypeptides, and the arrangement of phospholipids in the cell membrane [49,50]. Nanotechnologies draw inspiration from this biological phenomenon, using molecular blocks to self-assemble into organized structures applicable to drug administration and biomedicine, sensors, circuits, and devices [51,52]. As their capacity can be adapted, the encapsulation and controlled administration of drugs in self-assembled structures should be more versatile [53]. This review demonstrates this capacity, particularly that of multi-arm copolymers.

The self-assembly of copolymers results in various types of aggregates, among the most interesting of which are micelles, vesicles, and polymersomes. Micelles are amphiphilic aggregates formed by amphiphilic block copolymers, in which the hydrophilic blocks come into contact with a polar solvent to form the corona, whereas the hydrophobic blocks form the core of the micelle. The size of spontaneously formed micelles generally ranges from 10 to 100 nm [54]. The structure and size of micelles depend on parameters such as the size of the hydrophobic domain, the size of the polar group, ionic strength, concentration, temperature, pH, and, more specifically, on the topology of the copolymer, which will be further discussed [55]. Depending on the topology, micellar structures can include sphere, lamella, worm-like, and disk shapes [56,57]. 

On the other hand, vesicles are another type of self-assembled system that were first reported just over twenty years ago by Eisenberg and colleagues [58], who observed polymer vesicles from the self-assembly of block copolymers of polystyrene-poly(acrylic acid) (PS-PAA). Initially, polymer vesicles were assumed to be out-of-equilibrium structures due to the glassy nature of the PS membrane, but their thermodynamic stability was subsequently established [59,60]. Further work confirmed that vesicular morphologies are not dictated by the kinetically-frozen glassy nature of the hydrophobic block because vesicles can be formed with low glass transition temperature (T_g_) hydrophobes such as Poly butadiene (PBD) and poly(propylene oxide) (PPO) [61]. Since these initial reports, hundreds of articles have described the formation of polymer vesicles, and a high number of review papers have been published [62].

Polymersomes and liposomes are types of self-assembled vesicles characterized by their hollow spherical structure and composition, consisting of phospholipids for liposomes/vesicles and sequenced copolymers for polymersomes [63]. The discovery of liposomes dates back to the early 1960s, when Alec Bangham first identified these structures as models for cell membranes [64]. Firstly, liposomes were employed for this purpose. However, in the 1970s [65], they were proposed as a straightforward drug delivery system. They offer an ideal solution for combination therapy, as they enable the simultaneous delivery of hydrophilic and hydrophobic compounds, unlike micelles, which can only encapsulate one type of compound [66,67]. Hydrophilic compounds are encapsulated in the hollow core, whereas hydrophobic compounds are loaded into the bilayer. The robust, stable, and tunable membranes, high loading capacity, and long blood circulation time of the liposomes further enhance their value for targeted delivery of a wide variety of therapeutic products with appropriate pharmacokinetics. Previous studies have demonstrated their effectiveness in this regard [68,69,70]. The characteristics of liposomes are controlled by various factors, such as the lipid composition, number of lipid bilayers, size, surface charge, non-polar chain length, degree of unsaturation, and preparation method. These properties are essential to defining the features and functions of liposomes in drug delivery applications [71].

Furthermore, block copolymer vesicles, known as “polymersomes”, exhibit superior mechanical and physical properties compared to lipid vesicles (liposomes). Studies have shown that polymeric vesicles are more robust, with a critical surface deformation before rupture ten times greater than that of lipid vesicles [72]. The thickness of the membrane increases with higher molecular weights of the copolymers that make up these vesicles, leading to stiffer nanoaggregates under bending. However, the membrane elasticity, which plays a crucial role in endocytosis of vesicles into cells, has been found to be relatively independent of molecular weight, with values similar to those of phospholipid vesicles [73]. This elasticity is dominated by the chemical nature of the membrane–solvent interface and is therefore related to the surface tension [72].

Most biomedical applications of block copolymer vesicles require the copolymer to have zero or very low toxicity. To meet this requirement, various biocompatible polymers such as PEO, PLA, and PCL have been tested [74]. PCL, in particular, is popular in this field due to its biologically inert nature and the slow hydrolytic cleavage of its ester groups under physiological conditions. Previous research on this copolymer has demonstrated its ability to generate vesicles, with a controlled drug release up to two weeks [75,76]. 

Combining drugs with different activities and administering them simultaneously to the same cell or inflammation area can enhance the effectiveness of the drugs and tumor cytotoxicity. Peptide-loaded PEO-PCL vesicles have been successfully used to deliver therapeutic agents to the rat brain by Pang and colleagues [77], resulting in the improvement of learning and memory impairments induced by scopolamine. Additionally, researchers have developed oxygen carriers for potential use in treating ischemic tissues, using PEO-PCL copolymer vesicles loaded with either human or bovine hemoglobin that demonstrate good oxygen binding and release [74].

## 3. The Advantages of Polycaprolactone for Advanced Polymer Architecture Formation

Since the discovery of the first synthetic polyesters in the 1930s, poly(ε-caprolactone) (PCL) has remained one of the most important and widely studied biodegradable polymers in various fields. PCL is formed by a saturated aliphatic polyester chain with repeating hexanoate units. This semi-crystalline polymer has varying molecular weights, typically ranging from 3000 to 800,000 g mol^−1^. At higher molecular weights, crystallinity is reduced due to chain folding, resulting in a crystallinity of 33% at Mw = 200,000 g mol^−1^, which can reach 70%. In addition to this variation in crystallinity, degradation by bacterial and fungal enzymes is one of the parameters that has highlighted the interest in PCL, making it particularly attractive for biodegradable material applications. In addition to the expected esterase degradation, there is also evidence of PCL sensitivity to enzymatic degradation by lipases [78]. It should be noted that several parameters affect the degradation of PCL by hydrolysis, such as the rate of crystallization and the number of carbon atoms in the chain. These factors are known to affect the hydrolysis rate of esters under physiological conditions, which decreases significantly as chain length increases. PCL chains exhibit an elastic behavior at room temperature due to their very low glass transition temperature, which can be below −70 °C. In addition, the relatively low melting temperatures of around 60 °C make PCL materials easy to manufacture or transform into highly structured forms [79].

If we examine the production of scientific research regarding PCL applications, we can find that PCL-based materials are increasingly being used as specific drug release vectors in cell culture, regenerative medicine implants, and drug release [80]. In addition, the rheological properties of PCL have proven very useful for studying the principles of crystallization of complex and poorly understood polymers [81]. 

Although it is possible to produce PCL by the direct condensation of 6-hydroxycaproic acid, the most common method for large-scale synthesis of high molecular weight and low dispersity polymers is the ring-opening polymerization (ROP) of ε-caprolactone. However, there are reports that have used enzymatic means to produce PCL with a molecular weight of up to 10,000 g mol^−1^. ROP polymerization chemistry of PCL largely mirrors that of other common lactones, and a great deal of research has been performed to optimize procedures for this polymerization.

**Figure 2:**
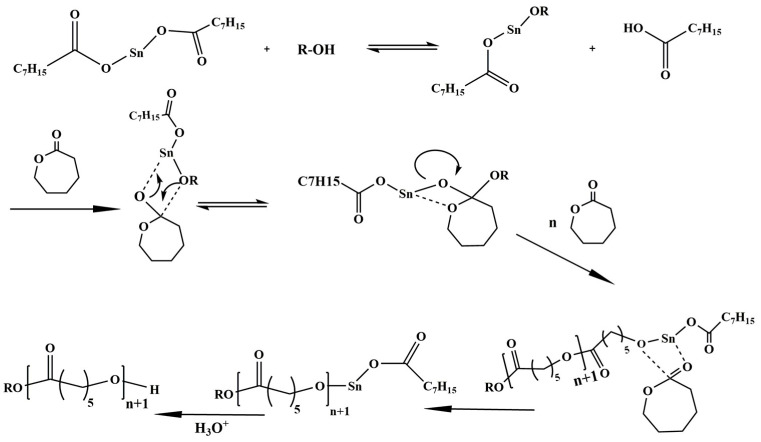
Synthetic routes to PCL: coordination–insertion polymerization, e.g., tin octoate.

The most commonly used method involves using a metal-based catalyst to polymerize ε-caprolactone through a coordination–insertion mechanism (Figure 2) [82,83]. Tin-based metal complexes (tin octanoate II) are particularly effective in inhibiting retro-inhibition and enabling the production of PCL chains with very high molecular weights up to 800,000 g mol^−1^ and a polydispersity of 1.1. These have certainly been widely used in ROP lactone polymerization chemistry. Obviously, PCL polymerization is a very rich field where efforts are being made to optimize reactions by using specific mechanisms with either organisms or transition metal-based or rare earth-based catalysts to increase yield and effectiveness. Synthetic methods for PCL have been more extensively covered in recent reviews [84].

The combination of orthogonal polymerization reactions, such as ring-opening polymerization, ATRP, NMP, RAFT, and click conjugation, has allowed for the development of complex polymeric architectures based on PCL. This has led to the observation of very attractive physical–chemical properties of this polymer. The PCL-b-PS arm polymers, which have an A_2_B_2_ structure emerging from a pentaerythritol core, have shown lower thermal transition temperatures and lower crystallinity than linear AB analogues, demonstrating that the characteristics of PCL can be changed depending on the architecture in which it is introduced [85]. The same was also observed in star-shaped block copolymers A_2_B, formed from a monoester of trimethylolpropane bromobutyrate, which has two free hydroxyl groups for caprolactone ROP and an initiator for ATRP [78].

In addition, one approach to accessing ABC-type multi-arm structures involves using a low molecular weight initiator that has a free hydroxyl group, along with an ATRP initiator branch (such as bromobutyrate) and an NMP initiator branch (such as TEMPO alkoxamine). This method allows for the introduction of PCL with various monomers. By growing each chain sequentially with PCL initiated by the free hydroxyl group in the ring-opening polymerization (ROP), multi-armed PCL can be produced over time [86].

Another way to access elegant architectures based on PCL is through the use of a combination of ROP and a six-armed dendrimer with alternating terminal groups free of hydroxyl and bromobutyrate, enabling the formation of multi-star arms. Moreover, PCL can be found in A3B3 structures based on PCL-b-PMMA and A3B-type structures based on PCL-b-PNIPAM, where the PNIPAM amine is coupled at the end through a combination of ROP and amide coupling [87]. The amphiphiles formed in this case are used as a basis for forming micelles in order to encapsulate hydrophobic drugs. The release of the drugs is thermo-sensitive above the LCST of the PNIPAM chains. Furthermore, another ABCD-type multi-arm structure was mentioned for the presence of PCL. The sequential polymerizations used in this case were ROP for CL, RAFT for MA, and click conjugation of a PEG chain to give the final multi-arm architecture PCL-b-PS-b-PMA-b-PEG [88].

These earlier studies that led to the development of nanoparticles with multi-arm PCL architectures have served as a foundation and model for subsequent work in the field. Various architectures have been produced, each exhibiting unique degradation, release, and stability profiles depending on the composition.

## 4. Application of PCL-Based Materials in Nanoformulations

One of the main goals of nanocarrier-assisted drug administration is to improve the quality of life of patients through effective therapeutic interventions while seeking better management and treatment of high-risk and high-morbidity diseases in a broader perspective. Oral, injection, or inhalation absorption does not meet these requirements. This is why controlled release systems have been developed to address the targeting problems posed by traditional administration methods. In this case, different formulations of sustained-release systems can be used to treat ocular diseases, such as gels, nanoparticles, microparticles, or implants. This requires a very comprehensive in vitro and in vivo biological evaluation of these nanomedicines to determine key parameters such as safety, distribution, specificity, accumulation, and elimination, which would help pave the way for clinical implementation. This section provides a brief overview of efforts in this direction, specifically focusing on the concept of PCL-based nanovectors. The process of clinical translation is quite complex, and the challenges can only be overcome through continuous and elaborate studies of various PCL-based polymer formulations, both in vitro and in vivo. These studies will provide necessary information regarding key physicochemical parameters, the structure/efficacy relationship, and the safety of star polymers and their final products after degradation or metabolism. The efficacy of nanoparticles is strongly related to their ability to reach targeted sites in the body, overcoming the enormous challenges of sustained blood circulation and cellular/intracellular absorption. 

Several studies in this field have provided a detailed overview of the factors that contribute to the improvement of the biological effects of nanocarriers. Upon introduction into the bloodstream, nanoparticles must be eliminated by the RES. In this case, previous research has shown that sustained-release systems are more effective in delivering drugs to targeted areas in the body, reducing the frequency of administration, decreasing toxicity, and improving patient compliance with treatment.

The problem of eliminating PCL-based materials, which have very slow degradation times by RES, has generally been solved by controlling opsonization, which depends on the physicochemical properties of nanoparticles. Despite this degradation problem, PCL has still been one of the most studied and applied biodegradable and biocompatible polymers in sustained-release systems. Additionally, PCL exhibits excellent mechanical and physical characteristics, as well as ease of processing and shaping at low temperatures. PCL has also been used in implantable devices for the sustained administration of various drugs such as antibiotics; antipsychotics; antiplatelet agents, namely dipyridamole and acetylsalicylic acid; nonsteroidal anti-inflammatory drugs (NSAIDs) such as ibuprofen; or even thyroid hormones such as levothyroxine. Another advantage of PCL is its compatibility with a wide range of drugs, allowing for the homogeneous distribution of lipophilic drugs in the support matrix due to its hydrophobic nature [89,90]. 

It has been shown that PCL does not cause any notable inflammation as it does not produce acidic products from biodegradation. The degradation mechanism of PCL follows the random cleavage of the ester chain group by hydrolysis and can take up to 2–4 years, depending on the molecular weight of the PCL. The higher the molecular weight, the longer the natural decomposition of PCL.

PCL-based materials have been studied for in vitro and in vivo biocompatibility and efficacy, and several PCL drug delivery devices have been approved by the FDA. When reviewing the biological and pharmaceutical literature that introduces PCL as a base material, in most cases PEG is present, which is commonly used as a crown for nanoparticles in aqueous environments. PCL-based polymers, essentially those combined with PEG, have been the subject of several studies dealing with the contribution of these nanoformulation polymers to better therapeutic efficacy and their translation into preclinical and clinical trials.

As already emphasized, different forms of PCL-based delivery systems, such as microparticles and electrospun mats, films, and scaffolds, have been developed to improve in vivo and in vitro effects.

### 4.1. Electrospun Mats, Films and Scaffolds

Fibers loaded with drugs were designed to deliver the drug to the targeted site in a sustainable manner without any burst release. Thanks to their fibrous structure, electrospun mats have a larger surface area than films cast in a solvent. Therefore, larger amounts of drugs can be delivered to the site where the mats are implanted. This was demonstrated in F. Annuryanti’s study [91], in which Triamcinolone acetonide (TA) was loaded into PCL-based implants using 3D printing techniques, which avoided the use of organic solvents. The development of biodegradable ocular implants capable of providing prolonged release of a corticosteroid drug, such as TA, for at least 6 months is a major advance in the fight against posterior segment eye diseases such as diabetic retinopathy. Drugs such as decorin (proteoglycan), mefoxin, and sodium cefoxitin retained their functionality after the electrospinning process [92,93].

Nanofibers of PCL encapsulated in ampicillin and coated with PCL have been developed using a diluted and partially electrospinnable PCL solution as the shell and a fully electrospinnable PCL solution containing a drug as the core. In another study described by Poornima et al., coaxial electrospun core–shell chitosan-PCL nanofibers containing folic acid (anti-inflammatory) and resveratrol (pro-angiogenic) were prepared for wound healing applications.

Star-shaped 6-arm copolymers (PCL-PEG) were synthesized with the aim of producing fibers that can be used in various applications such as clothing, medicine, sensors, and the automotive industry. The synthesis was performed with a discrete dipentaerythritol core that allows for the ROP of CL, followed by a Steglich esterification to attach PEG as the arms of this copolymer. To produce star-shaped block copolymer fibers, the electrospinning process was carried out under ambient conditions. The mechanical characteristics of the produced samples showed uniform and unique properties for micro-diameter fibers, which increases the fibers’ ability to trap drugs and can be used in clinical applications [94].

According to these results, poly (caprolactone) (PCL) has shown remarkable effectiveness for use as a biomaterial in scaffolds intended for tissue engineering, as well as for absorbable sutures, dressings, and anti-adhesion barriers, because it can adapt to different production methods. Materials containing PCL can be electrospun for the preparation of mats and can be used in various medical applications, including drug delivery [94]. Hsu et al. prepared electrospun PCL mats loaded with dexamethasone, which is used in the treatment of inner ear diseases and posterior segment eye diseases. Fibers with an average diameter of 300 nm and 670 nm were obtained. These hybrid systems allowed for the sustained release of dexamethasone for about one month [95].

In some cases, it is essential to modify the surfaces of fibers to improve drug attachment to nanofibers. For instance, to electrostatically bind doxorubicin to PCL, electrospun mats were partially hydrolyzed. Doxorubicin binding was achieved by immersing the hydrolyzed mats in drug solutions of different pH values. Drug absorption was high in basic medium, and drug release was high in acidic medium, indicating increased affinity for the tumor area [96].

In order to prevent rapid initial release under in vivo conditions, drugs were loaded into the core of coaxially electrospun nanofibers with a shell. Electrospinning can also be used to disperse particles and inorganic antimicrobial agents onto and into the surface of polymer fibers. When combined with clothing, these fibers can exhibit antibacterial properties. Therefore, electrospinning is promising for the development of membranes and fibers for protective clothing. Additionally, functional and intelligent fabrics can be made by blending two different materials and electrospinning composite fibers. These fibers can then be combined with a clothing membrane to create effective and practical materials. The fibers can be electrospun directly onto the fabric. Although this process overcomes the difficulties associated with sewing the electrospun membrane onto the fabric, the adhesion between the membrane and the fabric remains problematic. Several research organizations are studying plasma treatment strategies and chemical additives to improve membrane-to-fabric adhesion.

### 4.2. Hydrogels

Hydrogels are three-dimensional networks of hydrophilic polymer chains that can swell in hydrophilic environments without losing their structure and cannot dissolve in organic and inorganic solvents due to the presence of cross-linking between the polymer chains. From a rheological point of view, hydrogels exhibit viscoelastic behavior and partially purely elastic behavior [40]. In another aspect, the hydrogel has a very porous structure that permits substrates to be loaded into the gel framework and released at a rate that depends on the diffusion coefficient of the substrate through the gel network. In this concept different types of PCL-based multi-arm copolymers have been synthesized and used as physical hydrogels with different thermosensitive characteristics.

Multi-arm copolymers have been synthesized mainly by using a diol-initiated method such as PEG (with different numbers of arms). In this case, multi-block PEG/PCL copolymers with 3, 4, and 8 arms were synthesized using this method. At a certain concentration of the copolymer in the aqueous solution, the number of aggregates increases, and the volumetric fraction of the aggregates exceeds the maximum packing fraction, which leads to gelation of the system. Research has shown that the solubility of multi-arm copolymers in an aqueous medium is higher than that of linear polymers with the same hydrophobic/hydrophilic ratio. Additionally, PCL-based multi-arm copolymers form homogeneous and transparent hydrogels, whereas hydrogels formed from linear copolymers are generally white, opaque, and brittle, reducing their consistency during handling and limiting their applications in the field of encapsulation [97]. What is more impressive is that an increase in the number of PCL arms in the copolymers facilitates the aggregation and inter-aggregation of the bridges, thus facilitating the formation of the hydrogel [98].

The structure of PCL-based copolymers can influence the properties of the gel formed. PEG/PCL copolymers can be formed by an ester linkage between the PEG and PCL chains (PEG-OCO-PCL). The work of Buwalda and colleagues [99], who synthesized PEG-PCL copolymers with an amide bond between the chains (PEG-NHCO-PCL), showed that PEG-NHCO-PCL hydrogels exhibited a higher storage modulus (13 Kpa) than conventional hydrogels that contain ester bonds between the PEG-OCO-PCL chains (1.6 Kpa). This is due to the restricted conformational freedom of the amide groups, which leads to more effective physical cross-linking with a more rigid structure. In addition, interactions between the amide groups have caused an increase in the density of cross-linking and have improved the mechanical properties. In vitro studies have shown that the hydrolysis of PEG-OCO-PCL generally occurs at the ester linkages between PEG and PCL, resulting in a decrease in the hydrophobic chains of PCL and a disruption of the network structure. On the other hand, the amide groups in PEG-NHCO-PCL have been found to be more stable, and hydrolysis only occurs at the ester groups in the PCL chains. Thus, hydrogels formed from PEG-NHCO-PCL copolymers have greater in vitro stability (up to 16 days) than PEG-OCO-PCL hydrogels.

Despite these advantages, hydrogels have several limitations. Significant swelling of the hydrogel in aqueous environments leads to the formation of materials with poor mechanical properties that limit their use in load-bearing applications. Hydrophobic substrates such as drugs cannot be applied to hydrogels due to the thermodynamic incompatibility between the hydrophilic chain, water, and the hydrophobic drug.

### 4.3. Nanoparticles

Colloidal systems based on PCL are of great interest due to their use as drug carriers [100]. Self-assembled structures (micelles) have been widely studied as encapsulation agents for various drugs and proteins [101]. The use of nanocarriers for drug delivery arose from the need to find new vehicles for bioactive agents that would provide pharmacokinetic profiles that mimic the normal pattern of these agents, when the therapeutic efficacy of many of these agents is limited by the therapeutic index and selectivity [102].

Encapsulation methods improve and prolong the stability of bioactive drugs, enhance therapeutic efficacy by adapting the precise amount of drug to achieve the desired therapeutic response, and prevent degradation and nonspecific uptake by cells, thus minimizing side effects [103]. The structure of a transport system has a major impact on the loading of bioactive drugs and affects their release, cellular internalization, and in vivo biodistribution [104]. Micellar structures and micro- or nanoparticles of different morphologies allow for better control of degradation rates, release behavior, or drug distribution in the body [78].

An analysis of the literature, as well as these examples, show that a large part of the evaluation of multi-armed PCL-based polymers in nanoparticle formulation design has focused on drug loading and release, as well as in vitro studies on different cell lines, generally related to cancerous tissues. Some in vivo studies have also been conducted, but they remain few in comparison. For example, in amphiphilic polymer-based multi-armed micelles with dimensions of 10–100 nm, they are known to reduce protein-nanoparticle interactions and avoid nanocarrier elimination by phagocytosis [105]. 

To limit RES-based elimination and improve blood retention and circulation, copolymers can bind to drug molecules to increase their molecular weight, thereby reducing their elimination kinetics and increasing their effectiveness. The nanoparticles then follow passive pathways (enhanced retention by permeation) or active pathways (using targeting motifs) to reach the extracellular space. Accumulation at tumor sites depends on the camouflage, stability, blood circulation/retention, targeting ability of the nanoparticles, and other factors such as vascularization and angiogenesis [106].

Nanoformulations using multi-arm amphiphilic polymers help solve these RES-based elimination problems by modulating the density of the nanoparticle corona and facilitating the introduction of functional groups for active transport and drug release in response to stimuli. Cellular internalization of nanoparticles can occur through specific interactions with receptors, endocytosis, nonspecific association with the cell membrane, and absorption by pinocytosis [107]. Thanks to controlled and targeted drug efficiency, as well as their great ability to cross physiological barriers, biodegradable polymer nanoparticle-based delivery systems have shown sustained drug activity. Additionally, in addition to their reduced systemic side effects, drug-loaded nanoparticle systems reduce costs for patients and risks of toxicity. In this case, particle size is the primary parameter that influences targeting capabilities and penetration through physiological barriers.

PCL-based nanoparticles have generally been prepared using several techniques such as solvent evaporation-emulsification, nano precipitation, and solvent displacement methods, which provide richness in the operating modes used. During nanoparticle preparation, several parameters must be optimized, such as drug–polymer concentrations, homogenization speed and time, and types of solvents used. The effect of these parameters on the properties of curcumin-loaded PCL nanoparticles has been studied in the work of Kasinathan et al. [108]. In this study, PCL nanoparticles loaded with curcumin (with a diameter of about 390 nm) were prepared using the solvent emulsification and evaporation technique. The solvent emulsion-evaporation method was also used for intranasal brain delivery. PCL nanoparticles (about 300 nm) loaded with carboplatin (hydrophilic chemotherapy) were prepared for this purpose. In vitro cytotoxicity tests against human glioblastoma LN229 cells and in situ nasal perfusion tests conducted on rats showed better results compared to the use of a simple drug solution.

The RPE effect has become the most important mechanism considered in the design of cancer therapy. Nanocarriers are designed to take advantage of this RPE effect and accumulate in the tumor environment for better targeting and therapeutic efficacy. Vascularized, mainly metastatic, tumors of the pancreas, prostate, and liver are less sensitive to the RPE effect than other tumor types [6].

PCL-based micelles have multiple advantages, including improved permeation and retention (EPR), increased blood circulation time, and increased endocytosis due to surface modification. For the treatment of hepatocellular carcinoma, Chen and colleagues prepared lipiodol-loaded micelles using polyethylene glycol-polycaprolactone (PEG-PCL) copolymers and amphiphilic hyaluronic acid-polycaprolactone (HA-g-PCL) copolymers [109]. The HA-g-PCL micelles showed high drug encapsulation efficiency (about 71%), stability in aqueous solutions, and high affinity and cytotoxicity towards HepG2 cells, despite their size of 274 nm. 

The combination of drugs of interest with PCL-based formulations allows for non-covalent encapsulation of these drugs (based on hydrogen bonding interactions, hydrophobic-hydrophobic interactions, or ionic interactions) [110]. In this case, several factors such as drug solubility, drug affinity for the polymer, nanoparticle core volume, and drug self-aggregation capacity can influence the drug loading capacity (DLC), which is the mass ratio of drug to polymer. The DLC of paclitaxel in PEG-b-PCL-based micelles, for example, increases with the length of the hydrophobic PCL block [111]. On the other hand, no significant increase was observed when a similar experiment was conducted with doxorubicin, a less hydrophobic compound [112]. Furthermore, studies on the effectiveness of administration using in vitro cytotoxicity studies have also indicated that longer hydrophilic arms compromise the therapeutic efficacy of star-shaped supramolecular micelles charged with DOX, resulting in significantly reduced cytotoxicity, as measured by a higher IC50 value [113].

In addition to several experimental approaches, physicochemical models have been used to predict the charge and stability of the drug based on compatibility parameters between the polymer and the drug [114]. Among these models is the Flory–Huggins interaction parameter (ΧFH), which has been successfully used to characterize polymer-drug compatibilities [115]. Studies on PCL-based models have shown that drug loading is strongly influenced by the polymer composition and the shape of the nanoparticles [116]. “Worm-like” structures exhibit a higher packing density of PEG halves on surfaces with reduced curvature, leading to higher packing constraints for the polymers. Under these conditions, PEG chains extend, thus increasing the thickness of the outer PEG layer. Consequently, "worm-like" structures exhibit a drug loading twice that of spherical NPs. This variety of methods in the field of nanoparticles can facilitate screening of formulations and be used for the rational design of new polymers and drug-specific nanocarriers. It should be noted that the combination of a drug with a specific nanocarrier can have a significant influence on the pharmacokinetic profile and pharmacological effect of the drug [117].

“Intelligent” stimulus-sensitive nanoparticles could improve therapeutic effectiveness by enabling controlled drug delivery to the site of action. These formulations can be activated by external or internal stimuli, such as pH changes, temperature, irradiation, magnetism, concentration gradients, or enzymatic activity. Most cancer cells are found in an acidic environment, which stimulates the development of pH-sensitive drug delivery systems. This helps to reduce the harmful side effects of drugs used to treat cancer. Systems have been developed to respond to these stimuli and increase drug delivery to the target site [40].

Qi et al. prepared pH-sensitive copolymer micelles. These micelles, which measure approximately 100 nm, were obtained from a diblock copolymer of PEG and PCL linked by a pH-sensitive hydrazine (Hyd) bond, using a solvent evaporation method. The micelles were loaded with Paclitaxel (PTX), an anticancer drug. The results showed that these pH-sensitive and biodegradable copolymer micelles containing hydrazine exhibited strong toxicity against HepG2 cells [118].

In a different study, poly(ε-caprolactone) micelles grafted onto guar gum (GG-g-PCL) were prepared using a membrane dialysis method to load ketoprofen. Approximately 18% by weight of the drug was loaded into micelles ranging in size from 75 to 162 nm. Drug release was characterized by an initial rapid release, followed by sustained release over a period ranging from 10 to 68 hours [119]. 

Davoodi et al. [120] created amphiphilic graft copolymer systems of polyethyleneimine (PEI)-polycaprolactone (PCL) by combining non-viral gene therapy drugs with chemotherapy drugs to be used against aggressive cancers. They encapsulated the anticancer drug doxorubicin (Dox) and p53 plasmid DNA in positively charged nanoparticles (about 159 nm) with a hydrophobic core and a hydrophilic shell. The synergistic effect of Dox and p53 plasmid DNA showed better cytotoxic efficacy against human hepatoma and human cervical adenocarcinoma cell lines compared to their single-agent counterparts.

In another study, linear, 3-arm, and 6-arm star-shaped PCL-poly(quaternary ammonium salt) copolymers were synthesized using a combination of ring-opening polymerization and atom transfer polymerization. The hydrophobic PCL block formed the core, while the hydrophilic poly(quaternary ammonium salt) blocks served as the shell of the micelle. The antibacterial drug triclosan was loaded into these solvent-evaporated micelles. The efficacy of all types of micelles against *Escherichia coli* was reported, with star-shaped micelles showing better antibacterial properties [94].

Amphiphilic micelles of a multi-armed copolymer based on PCL/PEG and cyclodextrin developed by Xiufang Li et al. [121], have shown great efficiency in encapsulating DOX due to the adequate space present in this system. The in vitro release of the drug was studied using electrochemical control. In vitro release studies of DOX from star-shaped copolymeric micelles have shown that this anticancer drug can be released over several hours.

The ability of multi-armed copolymers to load and encapsulate a large number of drugs compared to linear analogues has been demonstrated in several other studies that have addressed drug delivery applications (Table 1) [122].

## 5. Exploring the Potential of Multi-Arm Polymers as Nanocarriers

The development of nanosized structures for drug delivery has strongly relied on linear amphiphilic block copolymers since the discovery of liposome nanoaggregates [134]. 

The last few decades have witnessed significant progress in the synthesis of these copolymers, and their self-assembling has been extensively studied, resulting in the creation of a library of functional groups that can be customized to improve drug solubility, loading, release, and bioavailability while reducing the required dosage [69]. Most of the current knowledge comes from studying their linear architectures. Detailed assessments of the behavior of self-assembled copolymers in aqueous environments have enabled the prediction of the resulting structure morphology. 

The hydrophilic/hydrophobic ratios in polymer compositions have been reported in the literature to be critical in producing assemblies with varying shapes and properties, such as star-like, crew-cut, and rod-shaped micelles, polymersomes, and lamellar structures [135,136]. Moreover, many papers have investigated the properties of their ordered assemblies, as well as the controlling factors, such as the chemical composition and molecular weight of the polymer [137].

The recent years have been crucial for the synthesis of star polymers due to the advances in the synthesis and characterization methods which give more control and more complexity to the fabrication of multifunctional polymers, such as polymers with symmetrical and asymmetrical stars and “Multi-arm” polymers [43,138].

The importance of branched copolymers in the nano-assemblies have encouraged many works to develop methodologies to obtain well-defined homo- or hetero-branched copolymers. For this, various radical polymerization methods have been employed, including atom transfer radical polymerization (ATRP), reversible addition-fragmentation chain transfer (RAFT) polymerization, stable free radical polymerization (SFRP), and ring-opening polymerization (ROP) [139]. These methodologies can be classified into three main approaches: core-first, arm-first, or grafting approaches [140].

The arm-first methodology involves using pre-synthesized polymer arms that are covalently linked to the core. "Click" reactions such as alkyne-azide and esterification have been particularly popular in this approach. In general, a combination of core-first and arm-first methodologies has resulted in the improved synthesis of amphiphilic polymers for self-assembly [141].

The increasing interest in branched copolymers as self-assembly nanocarriers has led to several studies focused on establishing a way to synthesize branched structures with the same composition as linear polymers [142]. To achieve this, varying the number of arms linked to the central core, along with controlling molecular weight, has been suggested as a means of providing flexible and less viscous branched copolymers [143,144,145]. Controlling the molecular weight becomes increasingly necessary for polymers that exhibit crystalline behavior, such as polycaprolactone (PCL), which exhibits an amorphous character as long as the polymeric chain is short, thereby enhancing hydrophobic interactions between the polymer and the encapsulated drug [146,147]. Another advantage of using the multiarms of block copolymers instead of their linear counterparts is the smaller hydrodynamic diameters that can be obtained, which result from the unique core–shell structure formed during microphase separation [148,149].

The use of a copolymer with multiarms provides a high number of functional groups incorporated in the structure, which diversifies the application area of such copolymers, including but not limited to drug carriers [150,151], due to the unique physicochemical properties that enhance the efficacity of drugs, as well as thermoplastic elastomers [152], polyelectrolytes [153], hydrogels [154], and adsorption dye removal [155]. 

The synthesis of the new macromolecule family formed by branched copolymers typically involves controlling several factors, which will be further discussed in this review, to modify their dimensions, morphologies, stability, and material properties. The next sections will discuss the strategies previously reported for designing self-assembled PCL-based copolymers for drug delivery purposes.

## 6. Impact of Multi-Arm Topology on PCL-Based Polymer Properties for Drug Delivery

**Figure 3:**
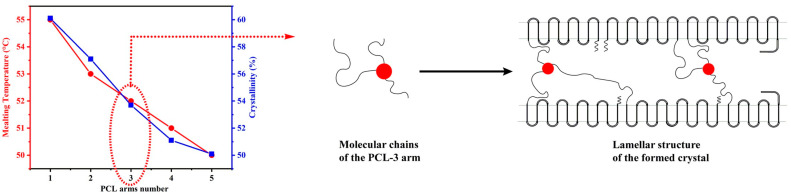
Influence of Arm Number in PCL Star Polymers on Melting Temperature, Crystallization Temperature, and Degree of Crystallinity.

PCL star polymers synthesized through ring-opening polymerization using multifunctional initiators have been extensively studied due to their potential for various applications such as investigating thermal behaviors, unimolecular micelles, and as the polymer network components. Wenyuan Xie’s paper [156] demonstrated that increasing the number of arms in multi-arm structures of PCL star polymers leads to a decrease in melting temperature, crystallization temperature, and degree of crystallinity. Furthermore, PCL star polymers with more branches have slower crystallization rates despite having higher molar mass, contrary to the behavior of linear chains, where higher molar mass results in increased crystallinity (Figure 3).

Other thermal studies on star-shaped PCLs with 8 arms [157], 6 arms [158], and 7 arms [159], as well as on star-shaped PCLs with 8-arms [160] and silsesquioxane (with an average of 30 arms) [161], have shown the same tendency of lowered melting temperatures. 

In addition, star polymers exhibit significantly improved thermal stability when compared to linear polymers of the same molecular weight. 

In this regard, recent work revealed that the crystallization rate of PCL (X_c,PCL_) reaches a maximum value of 65.3% for the linear polymer, with a subsequent decrease observed as the number of PCL arms increases. The order of crystallinity was found to be as follows: X_c,LPCL_ > X_c,3SPCL_ > X_c,4SPCL_ > X_c,6SPCL_ [162].

It was noted that the degree of crystallinity of the host polymer matrix seemed to be an important parameter because higher drug release rates were observed in polyesters with a low degree of crystallinity [163]. Evidently, several studies have shown that the degree of crystallinity of the copolymers directly influenced their apparent enzymatic degradation and, consequently, their efficiency to inhibit the drug target [164]. 

These fabulous results observed with multiple-armed macromolecular chains have laid the groundwork for the development of more complex architectures that are based on variations in arm structures. One such architecture is the miktoarm, or "mixed arm", which will be discussed in detail next. 

According to the literature findings (summarized in Table 2), these architectures exhibit lower thermal transition temperatures and lower crystallinity than linear analogues.

The development of multi-arm polymers is a subfield of PCL-based macromolecules that has garnered significant attention. The combination of ring-opening polymerization with other polymerization methods, such as ATRP, NMP, RAFT, and click conjugation, has enabled the creation of intricate structures [173,174,175]. As a result of the flexibility of these combined techniques, a diverse array of architectures can be obtained.

The work of Greg G. Qiao [176] and J. Du and colleagues [177] facilitated the first experiments that demonstrated the potential of multi-arm polymers in forming new types of nanoaggregates that were previously unseen with linear topologies, such as star structures with cross-linked cores (core cross-linked star). One of these works utilized PCL-PMMA, which was produced by linking homopolymers of PCL and PMMA. The variation in the length and arms of PCL led to the creation of a wide range of nanocapsules that can be degraded in a selective manner based on the length of the PCL arm, something that was not achievable with linear architectures.

**Figure 4:**
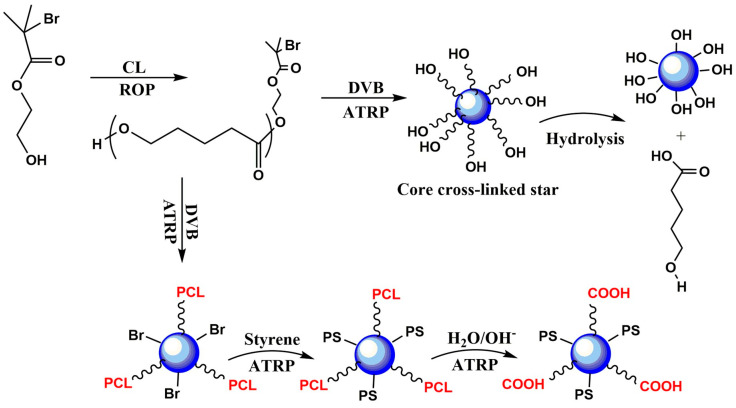
The potential of multi-arm polymers to form new types of core cross-linked stars and their eventual hydrolysis.

This has resulted in the use of these types of polymers as a base for the preparation of monodisperse nanoparticles. Similarly, the utilization of PCL-Br as a macroinitiator for ATRP, followed by its crosslinking with divinylbenzene (DVB), facilitated the development of active star centers for the polymerization of polystyrene (PS). The PCL chains of this multi-arm star could undergo biodegradation by hydrolysis under alkaline conditions (Figure 4). Besides being biodegradable, the micro-domain sizes present in these structures indicate potential benefits in terms of micelle sizes and properties.

The research conducted by Z. Zhuang [178] focused on the crystallization mechanism of a mixture of a four-arm PCL-PLLA copolymer and a PLLA homopolymer showed that the variation of the PLLA length in this multi-arm structure has a significant effect on the crystallization state of the mixture and that the degradation behavior is closely related to the crystallization state of the mixtures. This highlights the ability of the multi-arm to alter the thermal properties of the entire mixture, not just the character of its own structure.

Moreover, what is particularly remarkable is that, in addition to being able to regulate the thermal characteristics through the manipulation of the number or length of the arms in the copolymer, these topologies have demonstrated alterations in such features depending on the sequence of the chain. In the work of D. Deokar et al. [171], it was noted that when PLA was present as the outer block, the sequenced copolymer presented two T_m_ and T_g_, characteristic of each segment, one crystalline and the other amorphous. This proves the separation of phases in the 6s-PCL-b-PLA branched star with six arms. As for the 6s-PLA-b-PCL, or the PCL is the outer block, only one T_g_ was detected, which confirms the phase miscibility. This wealth of structure–physical property allows the copolymer arm to become a particular choice in various domains, especially for the formation of nanoaggregates that are performed by the separation of phase and thermal properties, such as the T_g_ and T_m_ of the copolymer.

The advancement in copolymer synthesis has resulted in the creation of alternative architectures known as “Y-shaped structures”. These special star-shaped polymers have garnered significant attention due to their one-of-a-kind morphology and phase behavior. 

**Figure 5:**
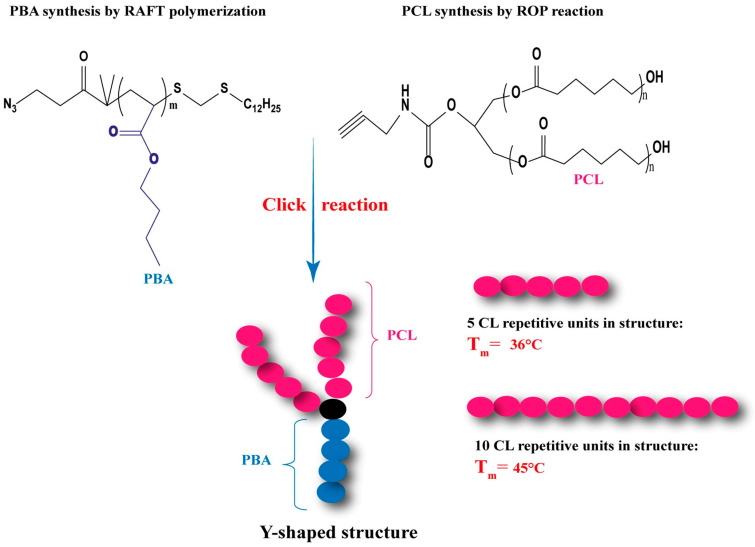
The Evolution of Melting Temperature (Tm) of PCL Blocks with CL Repetitive Units in Y-Shaped Structures.

Dean C. Webster et al. [166] were able to synthesize AB_2_ type star polymers (A: PBA) by using a combination of (RAFT), (ROP), and “Click” chemistry (Figure 5). Their PCL-based polymers have attracted particular interest in the field of controlled drug release. Those architectures have unique sol-gel properties, where they can be injected in the form of a solution and form gels at body temperature. Analysis shows that the melting temperature (T_m_) of PCL blocks increases with the number of CL repetitive units per hydroxyl. The obtained multi-arm polymers are crystalline because the T_m_ value of the polymers is higher than room temperature. This has also been observed in several studies such as the A_2_B_2_ multi-arm polymers of PCL-b-PS radiating from a pentaerythritol core, which have shown lower thermal transition temperatures and lower crystallinity compared to their linear architecture [78,179]. 

Varying the length of the PCL arm not only influences the thermal properties of the copolymer but also impacts the characteristics of the resulting nanoaggregates. It was found by the group of O. Glaied [180], who presented an elegant methodology for generating multi-arm stars based on (PCL)_2_-PS and (PCL)_2_-PMMA, that changing the length of the PCL arm chain allowed for the modification of the thermal stability of the micelles and also for the modulation of their size during self-assembly, which is of great interest in the field of nanoaggregate formation.

**Figure 6:**
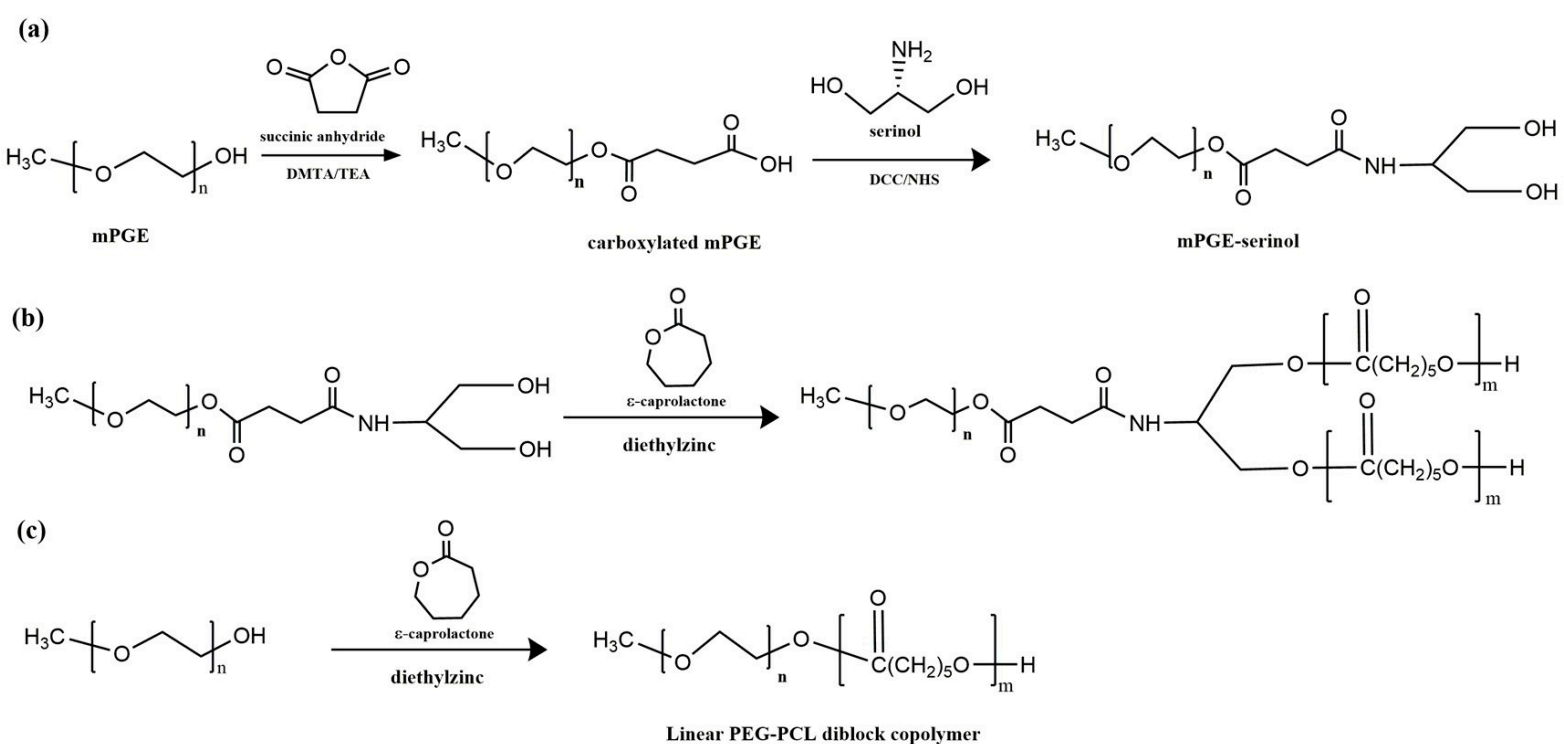
The AB_2_-type miktoarm copolymer (PEG-PCL_2_) synthesized using a “core-first” strategy (**a**,**b**); Linear PEG-b-PCL diblock copolymers synthesize (**c**) (Adapted from [181]).

Another promising result was also achieved by YH Bae and colleagues [181]. The AB_2_-type miktoarm copolymer (PEG-PCL_2_) was synthesized using a "core-first" strategy, which involves functionalizing the end of the mPEG chain, leading to the formation of two initiation centers on the mPEG macroinitiator for PCL arm growth. Polymerizations were carried out with different equivalents of the CL monomer to give a series of multi-arm copolymers with different PCL lengths. Linear PEG-b-PCL diblock copolymers, with a composition and overall molecular weight similar to that of miktoarm PEG-PCL_2_ copolymers, were also prepared to better understand the structure–property relationship between multi-arm copolymers and their linear counterparts (Figure 6). 

The structures based on multiarms demonstrated a superior capability compared to micelles formed from linear block homologues. Additionally, multi-arm micelles loaded with the DOX molecule exhibited a reduced release rate, increased cellular absorption, and improved cytotoxic effects compared to linear copolymer micelles. This finding indicated that multi-arm structures are more suitable than other morphologies for the efficient administration of drugs. The relationship between topology and its effect on self-assembly properties will be discussed in other sections.

**Figure 7:**
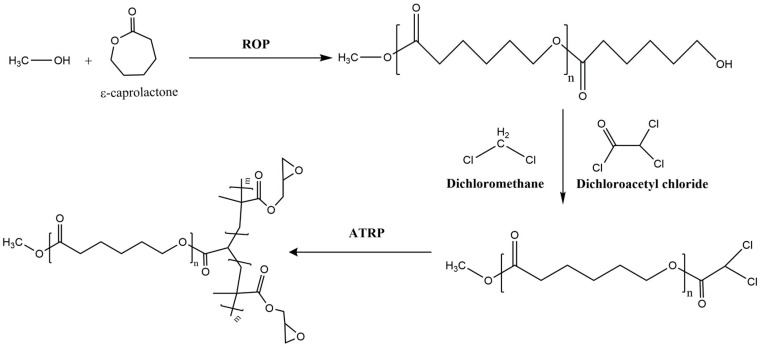
Synthesis of multiarms (PCL-PGMA) through the ROP and ATRP.

Modifying the composition of the copolymer in a multi-branched topology can lead to a more significant effect, namely a variation in the types of nanoaggregates formed depending on the structure. Yapeng Li et al. [182] demonstrated this by synthesizing new sequenced copolymers using a dichloro-polyester, which was initially synthesized through the ROP of PCL, as a macroinitiator for GMA polymerization by ATRP (Figure 7). The combination of these two processes yielded well-defined copolymers across a broad range of compositions. It was discovered that the PCL:PGMA ratios exerted a significant influence on the properties of the copolymers, particularly their micellization properties. These sequenced copolymers self-assembled into various morphologies, such as normal spherical micelles, vesicles, and lamellae. Moreover, it was observed that the copolymer composition and its concentration in THF significantly impacted the aggregate morphologies.

**Figure 8:**
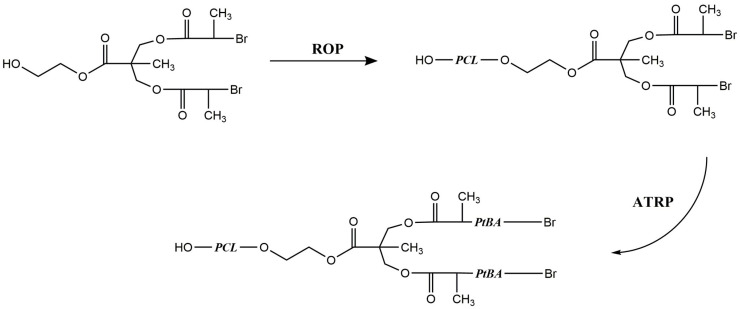
Schematic synthesis of A_2_B-type star polymers based on PCL-PtBA.

Through a three-step reaction sequence [183], a multi-functional initiator has been synthesized which has one initiation site for ROP and two initiation sites for ATRP. Initially, the initiator was used for the ROP reaction of caprolactone that yielded a polymer with secondary bromide end groups. Later, the polycaprolactone produced was used as a macroinitiator to carry out ATRP of tert-butyl acrylate (tBA) or methyl methacrylate (MMA), which resulted in the formation of A_2_B-type star polymers (Figure 8). The multi-armed polymers developed in this study exhibited a single transition temperature similar to that of the PMMA or PtBA homopolymers, likely due to the relatively short length of the PCL chains compared to those of PMMA and PtBA. As a result, the thermal transitions of the copolymers are dominated by the longer PMMA or PtBA chains. Although this reduces the thermal character of the PCL, it was observed that molecular weights are controlled with low polydispersity (weight-average molecular weight/number-average molecular weight ratio of 1.23) thanks to the ROP-ATRP sequence. This offers the possibility to modify the thermal behavior by altering the length of the PCL chains relative to those of PtBA or PMMA.

**Figure 9:**
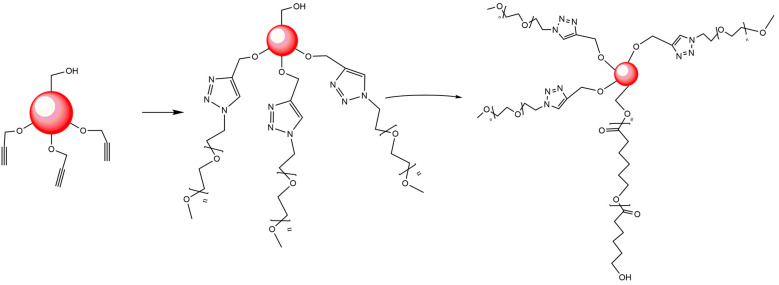
Synthesis of AB_3_-type multi-arm polymers based on PCL-PEG.

Other topologies, such as the AB_3_ structure, have benefited from the advantages of the AB_2_ and A_2_B structures. In 2015, a team led by Alexandre Moquin [184] presented a simple method for synthesizing AB_3_-type multi-arm polymers with one PCL arm and three PEG arms of varying chain lengths (Figure 9). The method combines ring-opening polymerization and alkyne-azide click chemistry. These AB_3_ miktoarm stars form micelles that efficiently incorporate curcumin, leading to a significant reduction in glioblastoma cell viability in spheroids. Asymmetric AB_3_ multi-arm polymers are easy to construct, providing a versatile structure for delivering various lipophilic drugs. Although oral drug delivery systems (DDS) based on amphiphilic linear polymers that form micelles have been extensively studied in recent years for poorly water-soluble drugs, several of them have not shown satisfactory performance, such as high critical micelle concentration (CMC), low loading efficiency, and uncontrollable drug release, which may lead to severe toxicity to normal cells due to high local drug concentration. This has driven the development of a new polymer design with a reasonable structure that could be an effective means to increase release efficiency.

In this concept, Lu-mei Huang et al. [185] explored a new pH-sensitive multi-arm sequenced copolymer for the delivery of hydrophobic drugs to improve the performance of the support for oral DDS. This group prepared new pH-sensitive micelles from a multi-arm miktoarm star sequenced copolymer, mPEG-bP(MMA-co-MAA)_2_, via an ATRP technique and dialysis method. The self-assembly of these miktoarms exhibited a very low CMC, indicating high colloidal stability in aqueous solution. MTX was used as a model drug with low solubility. The micelles showed high drug loading capacity, and 26% of the drug was released in simulated gastric fluid (SGF, pH 1.2) over 48 h, whereas the maximum drug release was 98% in simulated intestinal fluid (SIF, pH 7.4). Therefore, pH-sensitive micelles can be used as an effective carrier for the oral administration of hydrophobic drugs with prolonged and controlled release.

**Figure 10:**
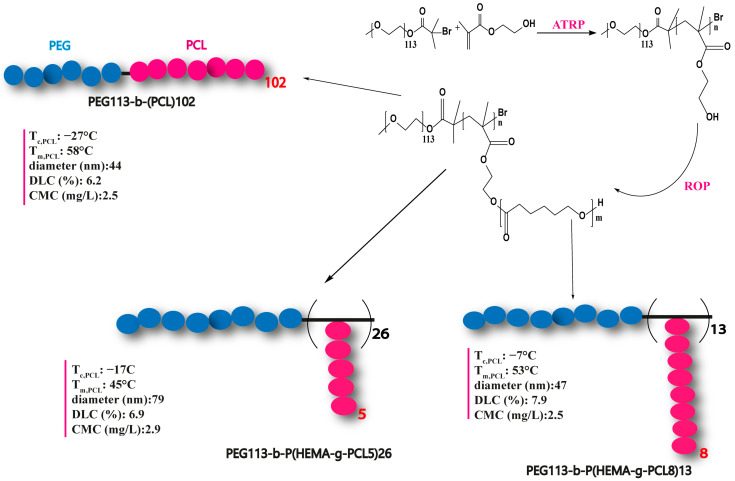
Synthesis of Amphiphilic Copolymers PEG-b-P(HEMA-g-PCL).

In the study by W. Zhang et al. [165], another multi-arm structure was described in detail and named toothbrush-type amphiphilic copolymer. This structure was based on PEG and PCL chains, where the number of PCL arms was represented by n in the formula PEG-b-(HEMA-g-PCL)_n_. These polymers were synthesized using a combination of ATRP and ROP, in which the macromolecular initiator of PEG-b-PHEMA initiated the ring opening of ε-caprolactone (CL). This multi-arm structure exhibited very different thermal characteristics compared to its linear counterpart. Furthermore, the increase in the number of PCL arms further reduced the crystallization temperature T_c,PCL_ of the toothbrush-type copolymer to a remarkably low value (−17.4 °C). These results suggest that the crystallization properties of these PEG-b-(PHEMA-g-PCL) toothbrush-type copolymers can be easily adjusted depending on the number and length of the PCL segments (Figure 10). The group also showed that these modifications of physical properties have an impact on assembly and encapsulation properties.

Due to the difficulty of recrystallizing the short and grafted branches of PCL under administration conditions, the micelles prepared from these toothbrush-shaped copolymers exhibited a higher loading capacity for DOX than the micelles formed by the linear polymer PEG-b-PCL. DOX-loaded toothbrush-shaped copolymer micelles also demonstrated exceptional stability in aqueous solution compared to DOX-loaded linear PEG-b-PCL polymer micelles. The in vitro release of DOX from brush-shaped copolymer micelles was faster than that of linear sequenced copolymer micelles. In addition, the studies revealed that DOX-loaded brush-shaped copolymer micelles could be effectively internalized by EJ bladder carcinoma cells and that DOX could be released in endocytic compartments and reach the nucleus. The brush-shaped copolymer micelles based on PEG and PCL, which have enhanced properties compared to those of linear PEG-b-PCL, can serve as versatile nanocarriers for a range of lipophilic drugs.

Another work conducted by V. Sathesh [186] utilized a simple and effective combination of ROP, SA ATRC (styrenics-assisted atom transfer radical coupling), and ATRP to synthesize PCL-based µ-star copolymers. The PCL-µ-PSt and PCL-µ-PtBA copolymers exhibited highly satisfactory physical characteristics. Additionally, the T_g_ of the amorphous segment and the T_m_ of the PCL segment were detected in both µ-star copolymers during the first DSC analysis, indicating the presence of semi-crystalline PCL. However, the T_m_ of PCL disappeared during the second scan, resulting from the restricted chain mobility of these polymers. Furthermore, a multistep degradation profile was detected, which was attributed to subsequent degradations of different arm analogues. The morphology of pure PCL demonstrated obvious heterogeneity due to the amorphous and crystalline macrophases, allowing these PCL-µ-PSt and PCL-µ-PtBA copolymers to form interesting nanoparticles with an average diameter of approximately 250 and 45 nm, respectively. The authors then performed a deprotection of the tert-butyl group from the PCL-µ-PtBA copolymer, and their studies showed a flat and smooth surface, which was due to the good miscibility of PCL and PAA segments. This highlights the ability of multiarms to change their physical properties through simple chemical modifications.

The impact of PCL arms on the properties of copolymers, whether in solution or in a solid state, has been determined in several other more complex architectures, such as grafted polysaccharides. The group of A. Ricardo et al. [172] demonstrated this effect on a lignin branched by PCL arms. The competition between lignin nucleation and PCL-g-lignin intermolecular interactions determines the crystallization behavior of PCL-g-lignin. The results presented in this work illustrate how the thermal properties of PCL can be adapted by grafting it onto lignin, which has a high potential as a renewable aromatic building block. Other specific properties such as antioxidant, antifungal, and fire resistance properties would also be an important function of the architecture and composition of these copolymers.

## 7. The Key Parameters of Self-Assembly in Multi-Arm Polymers

### 7.1. Solvophobic/Solvophilic Balance

All linear or multi-arm amphiphilic copolymers are capable of self-assembling into nanoscale structures in the presence of a selective solvent, due to their dual nature. Indeed, amphiphiles are, by definition, made up of solvophobic and solvophilic parts that interact in diametrically opposed ways with a selective solvent. Whereas solvophilic parts maximize their contact with solvent molecules, solvophobic parts minimize this contact. The balance between these two interactions leads to the formation of specific supramolecular structures, whose architecture is finely controlled by the solvophobic/solvophilic molar ratio. Using simple geometric considerations, we can define a critical packing factor as: Cpp = v/a × l [187].

Where (v) and (l) are the molecular volume and the length of the solvophobic parts, respectively, and (a) is the optimal surface per molecule occupied by the solvophilic chains in the final assembly.

**Figure 11:**
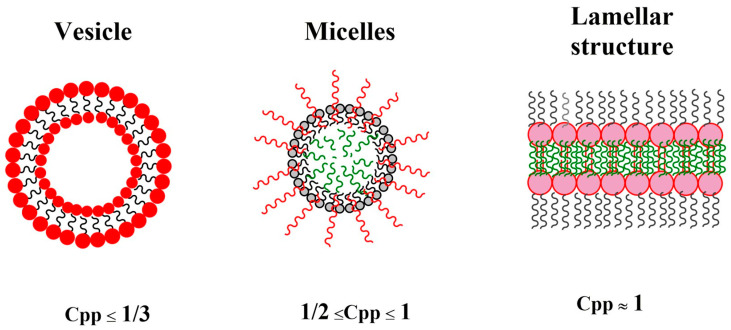
Variation of Critical Packing Parameter (Cpp) with Nanocarrier Morphology.

Generally, structures with high curvature (such as spherical micelles) are formed when Cpp < 1/3, cylindrical micelles are formed when 1/3 ≤ Cpp < 1/2, and low curvature bilayer membranes are formed when 1/2 ≤ Cpp < 1 (Figure 11).

In particular, amphiphilic block copolymers are the most useful, as the final structures can be finely controlled by the synthesis procedure. For amphiphilic block copolymers, the final packing factor is highly controlled by the ratio of hydrophobic and hydrophilic blocks. For example, experimental evidence has shown that for PEG-based systems, copolymers form spherical micelles with a PEG volumetric fraction (f) > 50%, cylindrical micelles with 40% < f < 50%, and vesicles with 25% < f < 40%.

**Figure 12:**
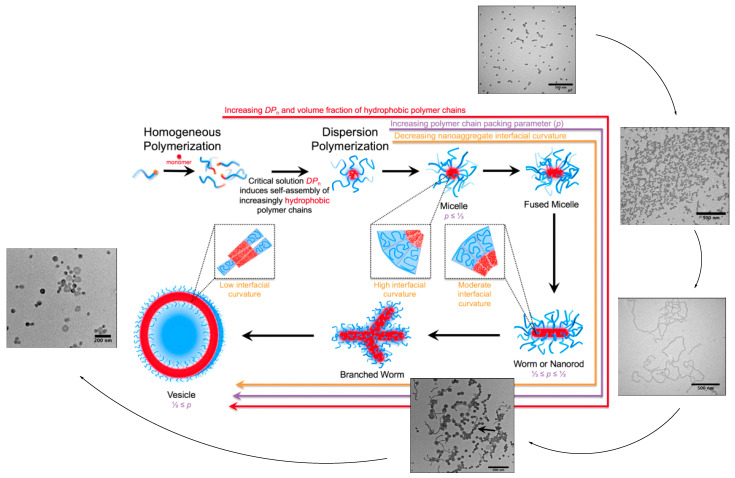
Polymerization-induced self-assembly process proceeding through a dispersion-based mechanism; Adapted from [188] (Copyright 2017, American Chemical Society).

In 2017, S. Sumerlin et al. [188] conducted a significant study in which they demonstrated how the hydrophobicity of amphiphilic block copolymers allows for the control of the morphology of aggregates in water. This was achieved through RAFT dispersion polymerization, and they reported that the morphology of the nanoparticles is defined by the growth of the hydrophobic ratio during dispersion polymerization. In addition, predetermined morphologies can be targeted by controlling the degree of polymerization (DP) of the hydrophilic and hydrophobic monomers during synthesis. Therefore, this elucidation of the contributions of the hydrophobicity of block copolymers during self-assembly can provide new perspectives and powerful strategies for programming the morphology and dimensions of block copolymer aggregates in solution. Micelles, worms, and vesicles have been synthesized, and the highest control levels over worm elongation achieved during polymerization were reported, simply due to the hydrophobicity of the polymer chain (Figure 12).

Applying the universal approximations previously mentioned is challenging for multi-arm polymers, particularly due to the variable branching architectures they exhibit. [189,190]. 

A comparison between AB and AB_2_ polymers (A = PEG, B = PLLA) with variable hydrophilic volume fractions of PEG revealed that the self-assembly of multi-arm polymers into polymersomes is much more tolerant regarding the variation of volume fractions. The formation of polymersomes was observed for PEG volume fractions of f = 0.2–0.7, which is much broader than the range of 0.2–0.4 for the corresponding double-block linear counterparts. This study showed that multiarms favor the formation of polymersomes or vesicles in contrast to micelles, and in entirely different approximations than linear polymers [191]. 

**Figure 13:**
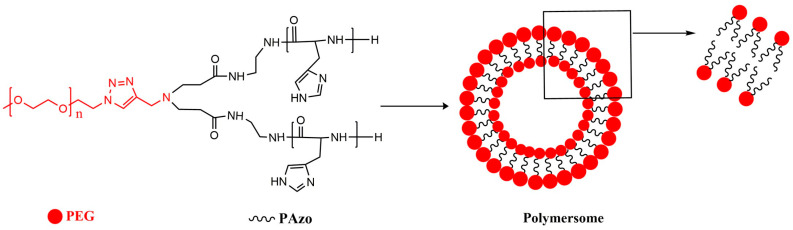
Representative Illustration of an Example of AB_3_ Polymersome based on PEG-PAzo.

In another example, multi-arm star polymer AB_3_ (A = PAzo, B = PEG) had a hydrophobic/hydrophilic ratio of 78/22, slightly outside the typical range for polymersome formation, but was nevertheless found to be well suited for polymersome assembly. Cryo-TEM micrograph analysis showed that the formed particles are polymersomes, due to the visual presence of thin outer membranes (Figure 13) [192]. 

**Figure 14:**
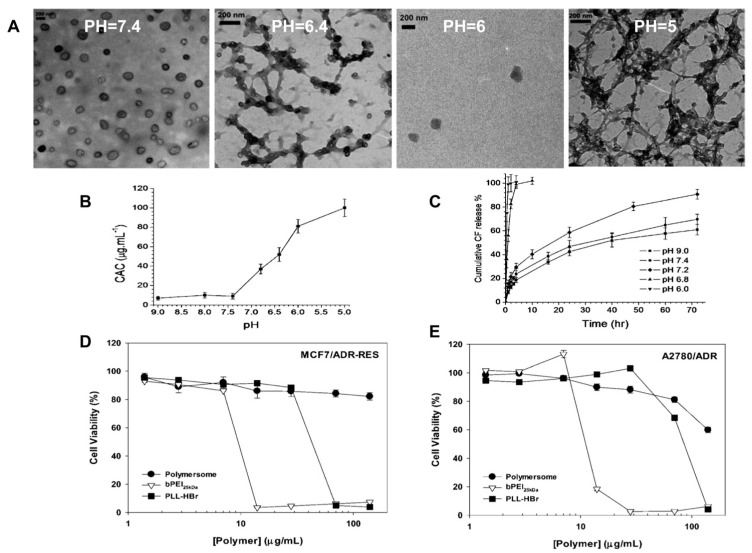
(**A**) TEM micrographs of the mPEG-b-(polyHis)2 polymer nanostructures at different pH. (**B**) The critical association value (CAC) as a function of pH. (**C**) In vitro release profiles of the encapsulated CF in the polymersome at different pH. (**D**,**E**) Cytotoxicity of polymersomes and representative polycations against MCF7/ADR-RES and A2780/ADR cells after 5 days incubation [192] (Copyright 2012, The Royal Society of Chemistry).

The polymer mPEG2kDa-b-(polyHis29kDa)2 is highly buffered in the endolysosomal pH range prior to acidification and exhibits low cytotoxicity after 5 days of exposure (Figure 14). Below pH 7.4, the polymer evolved from cylindrical micelles to spherical micelles and ultimately to unimers as the pH decreased. The pH-induced structural transition of mPEG2kDa-b-(polyHis29kDa)2 nanostructures may be due to an increase in the weight fraction of the hydrophilic portion of mPEG2kDa-b-(polyHis29kDa)2 and may contribute to the disruption of the endosomal membrane. Furthermore, the hydrophilic model dye 5(6)-carboxyfluorescein encapsulated in an aqueous polymer matrix exhibited slow and sustained release at pH 7.4 but highly accelerated release below pH 7.4, indicating the desired pH sensitivity of the system in the endosomal pH range [192].

The common point between both linear and multi-arm polymers is the fact that the length of the chains affects the morphology of the formed aggregates. In this regard, the work of Feihe et al. [192] has demonstrated that controlling the chain length ratio of diblock copolymers could influence the morphological transformation of aggregates. When the length of the hydrophilic PEO block chain is greater than that of the secondary hydrophobic block, micelles are formed. Under inverse conditions, vesicles are formed. Consequently, it is possible to control the chain length ratio of the blocks in solution, and therefore obtain various self-assembled morphologies with a controlled release function of the molecules.

**Figure 15:**
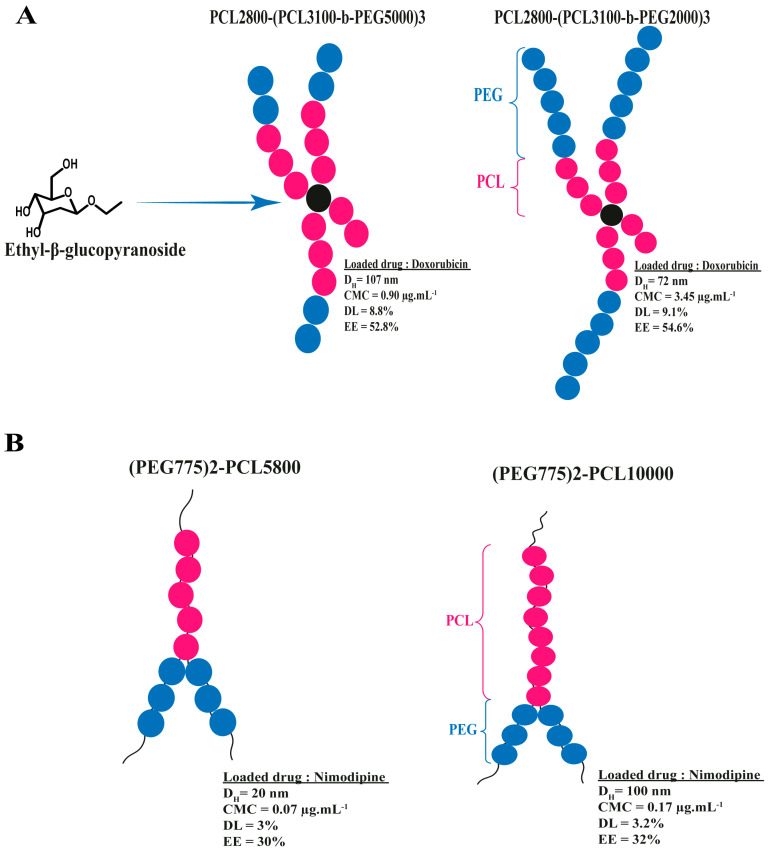
The effect of increasing the chain length on the nanoencapsulation parameters; for PCL(PCL-b-PEG)_3_ (**A**), and in the case (PEG)_2_-PCL (**B**).

Furthermore, the proportion of solvophobic and solvophilic chains could affect various properties, including the size of nanoaggregates, their stability, and the critical micelle concentration (CMC). Generally, an increase in the solvophilic components leads to higher CMC in aggregates. A study conducted by Zhu et al. in 2016 achieved the synthesis and characterization of an amphiphilic copolymer A(AB)_3_ polymer (where A = PCL_2800_ and B = PEG), which utilized an oligosaccharide as a branching core combined with PEG and PCL [193]. The resulting architectures exhibited relatively low CMC values that were lower than those of linear PEG-b-PCL copolymers possessing similar hydrophilic/hydrophobic ratios. In addition, the findings suggest that increasing the hydrophilic fraction of PEG in the multi-arm structure led to a decrease in the hydrodynamic diameter from 107 to 72 nm (Figure 15), with an increase in CMC from 0.90 to 3.45 µg.ml^−1^. The collected results could be attributed to the alteration in the hydrophilic/hydrophobic ratio, which affects the solvation/desolvation of PEG and PCL in the aqueous medium. The PCL_2800_-(PCL_3100_-b-PEG_5000_)_3_ system was more likely to aggregate, potentially due to its larger surface area and uniform shape. Furthermore, the micelles demonstrated strong biocompatibility, and they exhibited efficient drug transport properties towards cells [193].

The choice of polymer used to form the core of a nanocarrier can have a direct impact on the drug solubilization, loading capacity, and release kinetics of the carrier. In addition, the effectiveness of the encapsulation and the controlled release profile are directly affected by the size, morphology, and interactions of the copolymer blocks [194]. This is due to the polymer’s ability to interact with the drug through various mechanisms such as hydrophobic interactions, 𝜋-𝜋 interactions, and hydrogen bonding. When choosing a polymer for the core, it is important to ensure that the polymer is compatible with the drug being delivered. Polymers can be carefully selected based on their chemical properties and functional groups to optimize their interactions with the drug. For example, hydrophobic polymers could improve the solubilization of hydrophobic drugs by forming micelles in aqueous media. In addition, the length of the hydrophobic polymers could also affect the critical micelle concentration (CMC) of the carrier. Increasing the hydrophobic polymer length could decrease the CMC, which can highly enhance drug solubilization and loading capacity [195]. However, it is important to balance the hydrophilic and hydrophobic content of the polymer to ensure that the carrier remains stable in aqueous media. If the hydrophobic domains are too large, the micelle may have a larger core, which can lead to instability and aggregation. In order to achieve the desired drug delivery performance, it is important to meticulously optimize the selection of the polymer and its chemical composition.

In 2013, Y. Hyeok and et al. [196] synthesized a series of novel amphiphilic copolymers, including hydrophilic PEG blocks and hydrophobic PCL blocks. These multi-arm amphiphilic copolymers, which mimic the structural properties of phospholipids and have high potential domains, are capable of forming nanostructures and serving as effective drug delivery systems. DOX was chosen as a model drug to be loaded into the PEG-PCL multi-arm nanocarriers, and the in vitro release profiles of these DOX-loaded nanocarriers showed sustained release patterns. Additionally, PEG-PCL_2_ nanocarriers showed better cellular uptake. The authors of this study have shown that the length of the PCL arms directly influences the properties of the nanocarriers and that multiarms self-assembled into spherical micelles in aqueous solution when the length of the PCL blocks decreased. Similarly, a lamellar micellar structure was observed when the length of the PCL blocks increased.

In 2010, a report was published on a series of copolymers composed of (PEG)_2_-PCL_n_, where the value of n ranged from 3000 to 19,000 [197]. The micelles formed by (PEG_775_)_2_-PCL_19000_ had the largest hydrodynamic radius Rh, which was expected due to the longer PCL chain leading to a larger core. Additionally, this copolymer has demonstrated the lowest CMC and highest encapsulation rates. Linear diblock copolymers, PEG_2000_-PCL_5800_ and (PEG_775_)_2_-PCL_5800_, were also created, and it was discovered that the multi-arm structures resulted in smaller-sized micelles. Similarly, W. Lin’s [198] development of multi-arm polymers in aqueous solution resulted in exceptionally low CMC values (ranging from 0.0029 to 0.0035 mg mL^−1^), which suggests that the micelles have a high degree of stability in solution. The micelles, whether blank or containing doxorubicin (DOX), were able to self-assemble into spherical shapes with average sizes between 110 to 240 nm, depending on the copolymer architecture. These findings, as well as others, clearly illustrate the significant impact of polymer composition on micelles properties. 

On the other hand, Alizadeh et al. [199] have produced poly(ethylene glycol)-b-polyacrylic acid-b-polycaprolactone (mPEG-b-PAA-b-PCL) terpolymers with multiple arms that are sensitive to pH. They employed both the “click” reaction and single-electron transfer living radical polymerization (SET-LRP) to create these terpolymers. These multi-arm terpolymers assembled into micelles that had a PCL core, which is hydrophobic, and a PAA and PEG shell that is hydrophilic and released. The CMC value ranged between 1.40 and 4.46 mg.L^−1^, resulting in more stable micelles and a wider range of applications for controlled drug delivery. In comparison to linear triblock copolymers, these multi-arm copolymers exhibited lower CMC values, and an increase in PCL length led to an increase in drug loading (DL) and drug entrapment efficiency (EE). Furthermore, when administered intravenously or orally, these multiarms showed low cytotoxicity.

### 7.2. Linear versus Multi-Arm Copolymers

**Figure 16:**
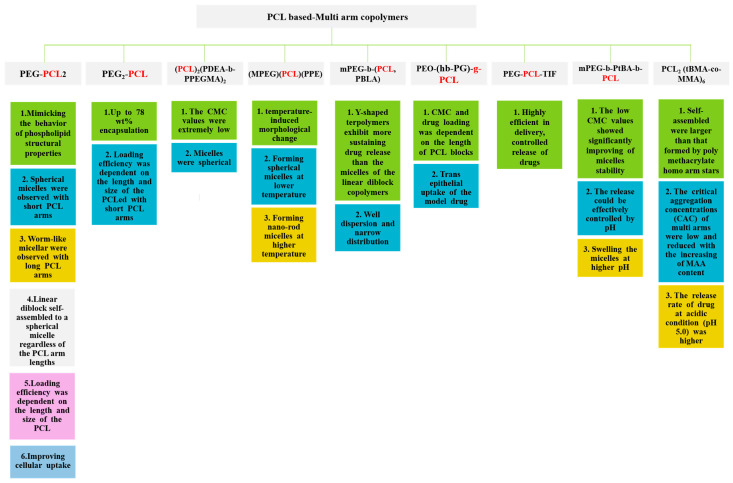
Ability of multi-arm architectures to respond to the various requirements of the drug delivery.

As previously discussed, the branched or multi-arm architectures offer interesting properties to enhance the effectiveness of polymeric nanocarriers as drug delivery vehicles. However, very few studies have sought to establish a direct comparison of the effectiveness of linear and multi-arm polymer architecture (Figure 16).

Scott M. Grayson’s [200,201] studies highlighted the ability of multi-arm sequenced copolymers to encapsulate hydrophilic compounds in a hydrophobic environment. This property could potentially allow their use as vectors for effective transdermal drug delivery. Although the limited number of arms (6 or 12) on amphiphilic sequenced star copolymers resulted in the formation of true unimolecular micelle properties, they showed improved encapsulation efficiency of polar dyes and reduced critical micelle concentration compared to linear analogues with only one arm. Furthermore, sequenced copolymers showed a notable improvement in transport. Multi-arm sequenced copolymer micelles also have a larger interior space, allowing them to contain more drugs. However, the majority of research on multi-arm polymers has focused on the synthesis of custom architectures and the relationship between structure and morphology [202].

**Figure 17:**
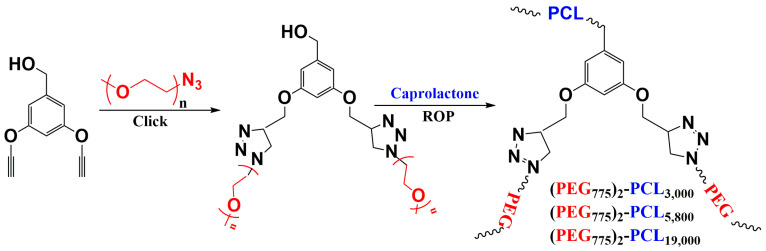
Controlled synthesis of miktoarm A_2_B copolymers with various PCL chain lengths.

Soliman and colleagues [197] have synthesized a series of miktoarm A_2_B copolymers consisting of a polyethylene glycol (PEG) A block and a polycaprolactone (PCL) B block for the delivery of nimodipine, a drug with low water solubility used to treat late-stage neurological ischemic disorders (Figure 17). These multi-arm copolymers (PEG)_2_-PCL generated micelles that displayed remarkable incorporation rates of up to 78%. Moreover, they facilitated sustained drug release, showed enhanced micellar stability, and exhibited reduced micelle size when compared to their linear analogues having a comparable composition and molecular weight.

**Figure 18:**
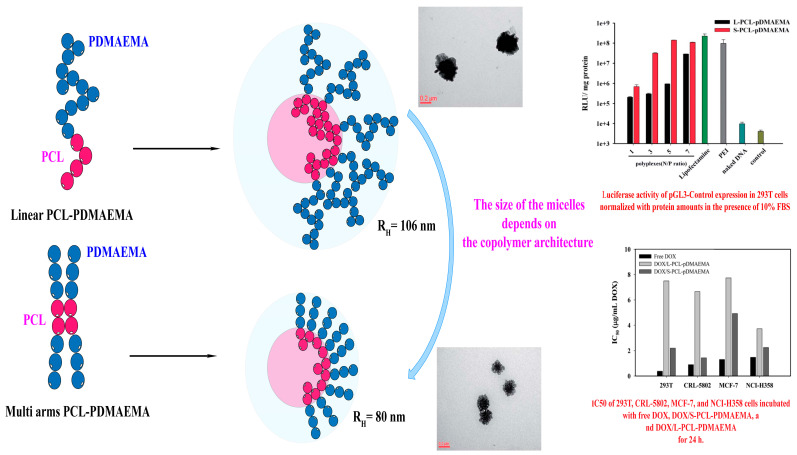
Schematic illustration of hydrodynamic radius changes as a function of PCL-b-PDMAEMA copolymer topology; Adapted from [203] (Copyright 2014, The Royal Society of Chemistry).

In 2014, Lo et al. [203] conducted a study comparing the synthesis and characterization of a four-armed copolymer (Star-PCL-b-PDMAEMA) and its linear analogue (Linear-PCL-b-PDMAEMA) of similar composition. The study aimed to evaluate their ability to deliver a plasmid and doxorubicin, an anticancer drug. The results demonstrated that the amphiphilic copolymers effectively transported both substances. When the copolymers self-assembled in a pH 3.0 solution, the branched copolymer micelles had a hydrodynamic diameter of approximately 80 nm, whereas the linear copolymer micelles were 106 nm in diameter (Figure 18). Additionally, the critical micelle concentration (CMC) of the four-armed copolymers (8.01 µg mL^−1^) was much lower than that of the linear analogue (21.2 µg mL^−1^). This was due to the finer cationic density resulting from the molecular weight distribution over the four arms of the star-shaped polymer, which led to the formation of smaller micelles. Although both the star-shaped and linear copolymers exhibited a very similar DOX charge (DL% 16.6 and 15.8%), the star-shaped polymer had a slower drug release profile and an increased cellular uptake, which was highly efficient in killing cancer cells. The S-PCL-PDMAEMA delivered drugs to the nuclei of U87 cells during a 3-hour incubation, but L-PCL-PDMAEMA accumulated most of them in the cytoplasm. In addition, the cell-killing effect of DOX/S-PCLPDMAEMA was better than DOX/L-PCL-PDMAEMA against 293T, CRL-5802, MCF-7, and NCI-H358 cells. This result showed that S-PCL-PDMAEMA presents a promising system for co-diffusion of therapeutic pDNA and hydrophobic anticancer drugs.

In the same area of study, Y. Bae et al. [181] found that the behaviors of the multi-arm PEG-PCL_2_, such as drug loading efficiency, self-assembly, and controlled drug release characteristics, differed from their linear diblock counterparts (PEG-PCL). The multi-arm copolymer with a high volume fraction of PEG (f_PEG_ = 0.55) and short PCL arm length self-assembled in an aqueous solution as a spherical micelle. However, cylindrical micelles were observed when the PCL arm length was longer (f_PEG_ = 0.15–0.32). In contrast, linear diblock counterparts (PEG-PCL) consistently self-assembled in aqueous solutions as spherical micelles, regardless of the PCL arm length. The cylindrical micelles of PEG-PCL_2_ with DOX as a model drug have demonstrated superior performance in terms of drug-loading capacity compared to the spherical micelles of the linear diblock counterparts. Additionally, DOX-loaded cylindrical micelles exhibited a slower release rate, increased cellular uptake, and improved cytotoxicity compared to DOX-loaded spherical micelles. The results indicate that DOX-loaded cylindrical micelles are more suitable for effectively administering drugs than other morphological forms.

Similarly, X. Zhang and colleagues [204] have successfully synthesized linear-hyperbranched amphiphilic block copolymers, mPEG-(hb-PG)-g-PCL, with multiple arms using a simple two-step ring-opening polymerization strategy. They also synthesized linear mPEG-b-PCL block copolymers with a similar composition to compare the effects of hyperbranched architecture. The comparative studies have revealed that multi-branched copolymers are more advantageous than their linear counterparts, mPEG-b-PCL, for use as drug delivery vectors. The hyperbranched amphiphilic block copolymers mPEG-(hb-PG)-g-PCL had a higher drug-loading capacity (DLC) and encapsulation efficiency (EE) compared to linear mPEG-b-PCL block copolymers. Moreover, the hyperbranched amphiphilic block copolymers exhibited more sustained and durable drug release behavior. Therefore, these multi-armed block copolymers have the potential to be favorable biomaterials for controlled drug release.

**Figure 19:**
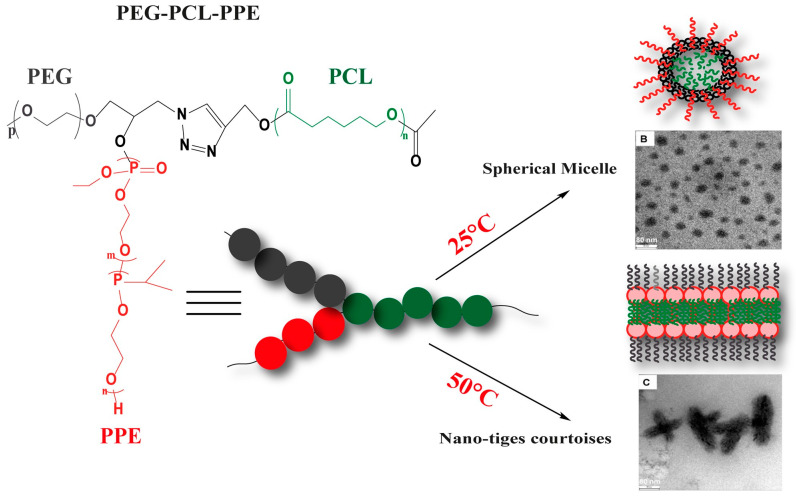
Ability of multi-arm copolymers to alter their morphology based on temperature; TEM analyses of ABC terpolymer assemblies in aqueous solution at 25 °C (**B**), and after heating to 50 °C for 72 h (**C**). Adapted from [205] (Copyright 2011, Elsevier).

However, in 2011, Yuan et al. [205] introduced a different type of multi-arm architecture. This alternative design utilizes an ABC terpolymer that changes its aggregate morphology in response to temperature fluctuations in an aqueous solution. The terpolymer, called a multi-arm terpolymer (MPEG)(PCL)(PPE), is composed of a hydrophilic segment made of monomethoxy poly(ethylene glycol) (MPEG), a hydrophobic section of PCL, and thermosensitive polyphosphoester chains that are linked by a junction site. When formed in an aqueous solution, the micelles created by this multi-terpolymer demonstrate thermal sensitivity. At low temperatures, the micelles are spherical in shape, but at higher temperatures, they transform into a nano-rod morphology once the cloud point is surpassed. The morphological transition of this miktoarm terpolymer caused by temperature fluctuations suggests its potential for drug delivery that is regulated and intelligent (Figure 19).

In 2014, a group of researchers led by Wenjing Lin [198] successfully synthesized (PCL)_2_ (PDEAEMA-b-PPEGMA)_2_ amphiphilic miktoarm star polymers by combining ROP and atom transfer radical polymerization (ARGET ATRP). The CMC values of these polymers were remarkably low (ranging from 0.0024 to 0.0043 mg mL^−1^). The micelles, both empty and DOX-loaded, were spherical with average sizes of 63 and 110 nm, respectively. The in vitro drug release was pH-responsive, with a faster release at pH 5.0 compared to pH 7.4 and 6.5, due to the protonation of DEAs’ tertiary amine groups. Intracellular uptake of DOX demonstrated its efficient delivery into cells after incubation with DOX-loaded micelles. Overall, the results suggest that pH-responsive (PCL)_3_ (PDEAEMA-bPPEGMA)_3_ polymeric micelles have the potential to be used as improved vehicles for cancer therapy.

### 7.3. Arm Number and Branching Degree

**Figure 20:**
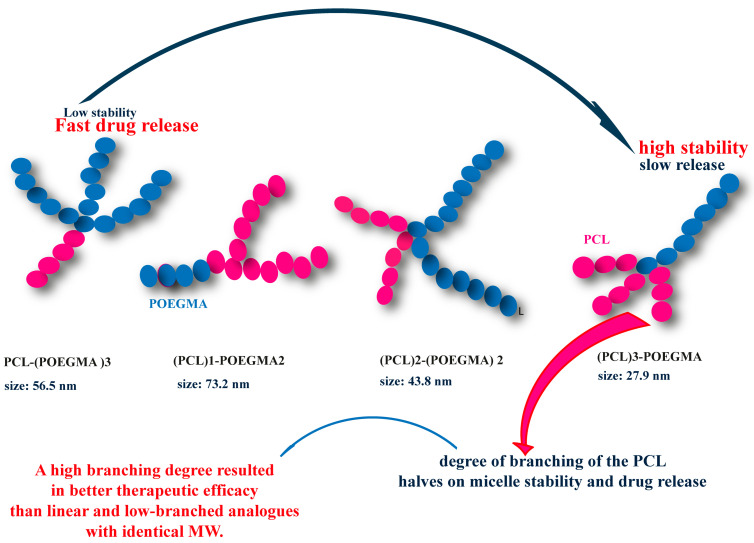
Structural Adaptation of Arm Number in Star-Shaped Copolymers (POEGMA-PCL) as a New Strategy for Designing Highly Effective Drug Delivery Carriers.

Another advantageous approach to reduce CMCs is adjusting the degree of branching in multi-arm amphiphilic copolymers, as this affects the packing of the monomers during self-assembly. For instance, Wang et al. [206] studied four multi-arm polymers of A_2_B, A_2_B_2_, A_3_B, and AB_3_ types with similar compositions, where A = PCL and B = polyoligo (ethylene glycol) methyl ether methacrylate (POEGMA). According to their findings, the three-arm polymer (PCL_2300_)_2_-POEGMA_12300_ had a larger hydrodynamic diameter (73 nm) and higher CMC (18.19 µg mL^−1^) than the other four-arm polymers (28-57 nm, 2.66–10.00 µg mL^−1^). Furthermore, they compared the effect of the degree of branching of the PCL halves on micelle stability. It is not surprising that the four-arm polymer (PCL_1600_)_3_-PEGMA_11900_ has the smallest hydrodynamic diameter (28 nm) and CMC (2.66 µg mL^−1^), likely due to the higher degree of branching of the hydrophobic core, resulting in a more compact core and thus a smaller size (Figure 20). Similarly, this same polymer also had the highest DL% (34.3%) and EE% (62.9%). 

Another study aimed at exploring the influence of different PLGA-PEG arms on DOX delivery was reported by Guilei Ma, et al. [207]. They successfully synthesized well-defined S-PLGA-PEG copolymers with different numbers of arms (3s/4s/6s-PLGA-PEG), which were utilized to investigate the relationship between the number of arms of PLGA-PEG copolymers and their micelle properties. The CMC value of st-PLGA-PEG copolymers decreased with an increase in the number of arms in S-PLGA-PEG copolymers, indicating that the formation of micelles becomes easier as the number of arms in copolymers increases. The size of DOX-loaded micelles and drug content also increased in the same order as the CMC. Among the three star-shaped copolymers (3s/4s/6s-PLGA-PEG), DOX-loaded S-PLGA-PEG micelles with four arms showed the highest cellular uptake and cytotoxicity, possibly due to the difference in size and distribution of PEG on the surface of S-PLGA-PEG micelles. Thus, the structural design of the number of arms from S-PLGA-PEG copolymers could provide a new strategy for the design of highly efficient drug carriers.

**Figure 21:**
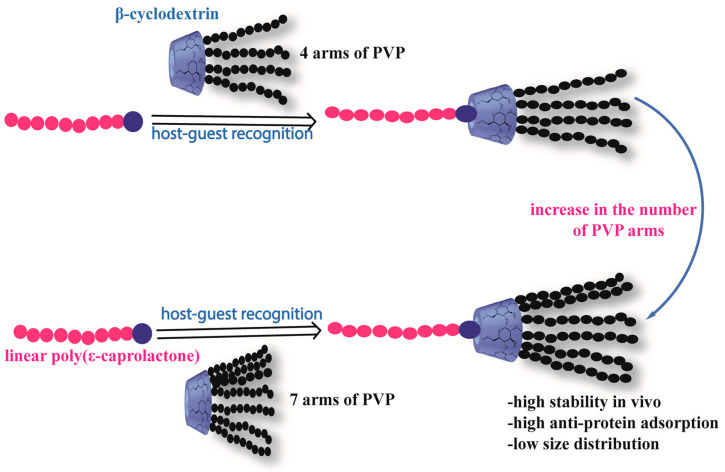
The effect of arm number on the encapsulation parameters of micelles formed from supramolecular structures based on β-cyclodextrin.

Drawing inspiration from the advantages offered by multi-arm topologies, new micelles formed from supramolecular structures based on β-cyclodextrin (β-CD) have been constructed [208]. These structures were linked to poly(N-vinylpyrrolidone) (PVP) chains with four and seven arms, terminated by a linear poly(ε-caprolactone). The micelles with PVP chains with seven arms on the outside showed the lowest amount of protein adsorption and the best stability in media, demonstrating the effect of the number of arms on encapsulation parameters. When cabazitaxel was loaded into the micelles, a drug content of 14.4% and an encapsulation efficiency of 85% were obtained. In vitro cytotoxicity studies showed that cabazitaxel-loaded micelles exhibited significant cytotoxicity against drug-resistant A2780/T cell lines. In vivo tumor inhibition experiments and survival rate observations demonstrated that PVP7-PCL-loaded micelles had superior efficacy in tumor inhibition compared to free cabazitaxel (Figure 21).

## 8. Degradation of PCL-Based Multiarms

The degradation of PCL-based products has been mentioned in several studies. PCL completely degrades in the presence of *Pseudomonas lipase* within 4 days [209], whereas hydrolytic degradation in the absence of enzymes takes several years [210]. This rapid enzymatic degradation of PCL highlights its importance in terms of biodegradation [211]. Only three types of lipases obtained from bacteria or fungi, namely *Rhizopus delemer* [212], *Rhizopus arrhizus* [213], and *Pseudomonas* [214], effectively accelerate the degradation of PCL.

When multi-arm copolymers with poly (caprolactone) (PCL) were subjected to degradation in a pH 7.4 PBS solution at 37 °C, it was observed that the degradation rate increased with a decrease in the arm length of PCL or an increase in the number of arms. Previous research has indicated that the enzymatic degradation of PCL is caused by random chain scission of the polymer backbone, regardless of the length of the chain [215].

According to reports, the enzymatic degradation of PCL is affected by the degree of crystallinity, with the level of crystallinity increasing during the initial stages of degradation, indicating that the amorphous regions are primarily broken down. Furthermore, it has been suggested that the degradation of PCL occurs mainly at the chain ends, folds, and crystal edges, where chain mobility is higher. This finding suggests that the type of architecture, including variations in the number of branches, can affect the degradation process [216,217,218].

**Figure 22:**
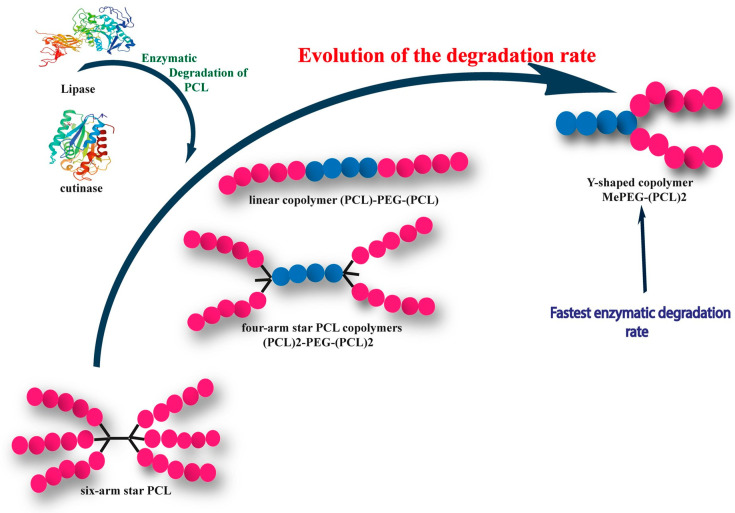
Impact of Architecture of Different PCL-Based Polymers on Degradation Profile.

In a work by Catherine J. Blackwell [219], a comparative study of degradation of different PCL-PEG-based architectures (Figure 22) is proposed. Four-armed PCL were connected to a PEG central unit (PCL)_2_-PEG-(PCL)_2_ (with different PEG contents), six-armed star-shaped PCL (PCL)_3_-b-(PCL)_3_ were compared with Y-shaped copolymers PEG-(PCL)_2_, and linear triblock copolymers PCL-b-PEG-b-PCL were subjected to enzymatic hydrolysis using lipase and cutinase. These studies showed an increased swelling and fragmentation in all copolymers due to the presence of PEG, leading to a faster loss of mass during enzymatic degradation using lipase. In contrast, the enzymatic degradation rate using cutinase generally decreased with an increase in PEG content in the case of PEG (PCL)_2_-b-PEG-b-(PCL)_2_, indicating that PEG chains slowed down the degradation effect of PCL in copolymers and that PCL was the only one subjected to enzymatic degradation. As mentioned earlier, this study showed that the %χc increased during the first two days of degradation, during which time mainly the amorphous regions were degraded. The second stage of degradation was followed by a decrease in crystallinity, as the PCL chains in the copolymers were degraded.

Significantly, the Y-shaped copolymer PEG-(PCL)_2_ showed the fastest enzymatic degradation rate by both cutinase and lipase enzymes. In fact, the slowest enzymatic degradation rate was observed in the case of the six-armed PCL copolymer (PCL)_3_-PEG-(PCL)_3_, which is due to its high hydrophobicity and relatively high molecular weight.

## 9. Limitations and Future Perspectives

The use of nanocarriers for drug delivery may be a promising strategy to effectively treat certain diseases. However, the development of nanocarriers for drug delivery is a complex problem that requires a multidisciplinary approach. Indeed, many factors must be taken into account, such as the size, shape, electrical charge, stability, and biodegradability of nanocarriers, as well as their ability to cross biological barriers such as the blood–brain barrier. Therapeutic polymers can also be used to specifically target certain cells or tissues, depending on their composition and size.

Overall, both nanocarriers and therapeutic polymers offer promising platforms for new drug development. However, the choice of platform will depend on the specific characteristics of the disease, underlying biological mechanisms, potential routes of administration, patient compliance, and other factors.

The creation of stable and safe drug formulations with improved bioavailability has led to advances in macromolecular precursors, moving from traditional linear block copolymers to amphiphilic compositions with multiple branches through chemical innovation. These polymers have unique structures that are highly useful for drug delivery. For example, their branched architecture minimizes chain entanglement, reducing solution viscosity and facilitating formulation administration. These polymers also offer a strategic balance through their hydrodynamic volume, terminal groups, flexibility, etc., which can improve their bioavailability and circulation in the bloodstream. Multiple branch compositions also exhibit low critical micelle concentrations, high loading capacity, and controlled release of the cargo. Additionally, functional chemical entities can be easily integrated into their architecture, making them very interesting for improved targeting and therapeutic outcome. These attractive characteristics have sparked increasing interest from the scientific community in these macromolecules.

The use of multi-arm polymers is becoming increasingly common to tackle various challenges, such as improving formulation stability, therapeutic index, surface functionalization, cellular interactions, and targeted delivery. However, there is still much to be done to establish their effectiveness and performance compared to linear block copolymers. Several variables of polymer-based self-assembly need to be studied, including polymer/drug ratio, compatibility, solvent choice, self-assembly method, and solvent/water ratio during formulation preparation. It is also important to examine the effect of pH and temperature on drug solubilization in star polymer assemblies, as this would establish the stability of micelles at appropriate pH and temperatures. Deepening our understanding of these formulations would be a good approach to improving optimal dosing and patient compliance.

Multi-arm-based nanoparticles can help with some of these issues, such as the difficulty in controlling the size and stability of linear copolymer nanoparticles and the need for more in vitro and in vivo research because of their ability to target the site of action, such as a tumor environment or infected cells. Another issue is the recognition of these nanoparticles by the immune system, which will lead to their elimination from the body that will significantly affect the drug release and transportation. The modification of the copolymer surface occurs in order to avoid their recognition as a non-self-composite, which will highly affect their drug delivery efficiency. Hence, these copolymers present a revolutionary way that will completely change how we treat illnesses by enhancing treatment efficacy and minimizing negative effects. To get beyond these issues and maximize the utility of copolymer-based drug delivery in clinical settings, more research and development is necessary.

Polymer chemistry specialists have developed methods to design multi-branched polymers that can be used for targeted drug delivery, imaging, and even precision medicine. The incorporation of targeting motifs allows for more effective retention through ligand–receptor interactions. Stimuli-responsive polymers offer a more direct approach to achieve on-demand release in response to the abnormal microenvironment conditions of cancerous tissues. Star polymers offer opportunities for intelligent self-assembly design through synthetic manipulation of the composition of the branched polymeric structure, facilitating accumulation and absorption at the site of disease for increased biodisponibility and therapeutic effect.

It is true that the direct comparison of formulations based on linear block copolymers with those based on star compositions is a crucial aspect that has not been sufficiently addressed in polymer research. Star compositions have potential advantages over linear block copolymers in terms of physical properties, stability, biodegradability, and functionalization.

However, the complexity of controlling all properties, such as overall and individual molecular masses of polymeric arms, hydrophilic fraction, etc., makes direct comparison between these two types of polymers challenging. Additionally, it may be difficult to generalize results from one study to another, as formulations can vary significantly depending on the synthesis method, reaction conditions, and monomers used.

Despite this, comparative analysis of the properties of linear block copolymers and star compositions is important as it can help determine which structure is best suited for a specific application. The results of such analyses can also provide further justification for the use of branched structures in the design of effective therapies.

In summary, although direct comparison between linear block copolymers and star compositions is challenging due to the complexity of controlling all properties, it is important to continue research in this area to determine which structure is best suited for a given application. The results of such analyses can provide further justification for the use of branched structures in the design of effective therapies.

## 10. Conclusions

In this review, an overview of multi-arm copolymers based on polycaprolactone synthesis, physical properties compared to their counterparts, and their interesting applications as nanocarriers have been highlighted. As described above, the transition from linear to multi-arm architectures came with many physical/chemical advantages such as low viscosities in solutions, low CMC, high loading efficiency, high density of internal/peripheral functionalities, and more, which has encouraged their biomedical applications over the past decade, especially with regards to drug delivery. The recent advances on their application as nanocarriers have been emphasized with respect to their various structures. It has also been established in this paper that a well-controlled polymerization processes is a key goal to prepare different forms of multiarms according to their intended applications, which enable better control of the biocompatibility, biostability, and biodegradability of the desired architecture. More developed multi-arm architectures are supposed to be revealed in the next few years following this trend and focusing on resolving the limitations of PCL-based multi-arm copolymers in drug delivery, more particularly in in vivo and in vitro studies.

## Figures and Tables

**Table 1:** The Biological Evaluations of PCL-based materials.

Mode	Polymers	Drug	Nanoformulation	Therapeutic Target	Biological Evaluation	Ref.
Electrospun	PCL	Dexamethasone	Nanofibers	Biomedical applications, particularly in the eye.	Although the results are encouraging, further in vitro studies and finally in vivo animal studies are needed to determine the comfort and retention.	[123]
Electrospun	PCL	Tetracycline/βcyclodextrin	Nanofibers	Controlled release systems and their clinical application.	Strong adhesion and reduced demineralization of dentin.	[124]
					High antimicrobial activity against A.a and P.g.	
Electrospun	PCL	ampicillin	Nanofibers		Zero-order drug release kinetics.	[125]
Electrospun	PCL	Seeded with IPFP and chondrones	Nanofibers	Restore the functional cartilage in articular disorders.	A positive effect on the differentiation of IPFP APCs into chondrogenic cells.	[126]
Electrospun	PCL-PEG	Fe_3_O_4_ NPs	Magnetic composite membrane (PCEC/Fe_3_O_4_ nanofibers)	Preventing tumor recurrence and improving dermal wound healing after an excision of malignant tumor in the skin.	In vitro cell culture of NIH 3T3 cells on the PCEC/Fe_3_O_4_ membranes showed that the PCEC/Fe_3_O_4_ fibers might be a suitable scaffold for cell adhesion.	[127]
					MTT analysis also demonstrated that the membranes possessed lower cytotoxicity.	
Electrospun	PCL-PEG		PCL fibers embedded in a PEG-fibrinogen hydrogel	Sufficient cell-approachable bio-signaling cues, which may synergistically facilitate the control of stem cell fates for regenerative therapies.	A novel nanocomposite that promoted the active interactions with stem cells and exerted excellent fibrogenic commitment in vitro.	[128]
Hydrogel films	PCL-PEG	Curcumin	Sol-gel	Safe candidate for in situ gel-forming controlled drug delivery system.	No toxic response or histopathological changes were observed.	[129]
					In vivo gel-formation, degradation test showed that a complete degradation occurred after 21 days.	
Hydrogel films	PCL-PEG	proteins (BSA and HRP)	Sol-gel	In-situ gel depot at body temperature providing drug release control.	The in vivo gel-formation and degradation studies indicated that copolymer hydrogels can sustain at least 45 days.	[130]
Hydrogel films	PCL-PEG		Sol-gel	Facilitate the bone regeneration in the non-load-bearing cranial repair process by combining the advantages of the intrinsic osteoinductive ABM granules and the injectable thermosensitive PECE hydrogel.	In vivo bone regeneration performance was carried out in white rabbits for 4, 12, and 20 weeks.	[131]
Hydrogel films	(PEG-PCL)_3_	cyclodextrin	Injectable hydrogels	Treatment of joint disease.	In vitro drug release showed that DOX∙HCl was released in a controlled, pH-dependent manner.	[132]
Nanoparticles	β-cyclodextrin-PCL	Camptothecin/DOX	Micelles	Sustained release of hydrophobic drugs via local administration in clinical situations	IND-M was able to release the drug over an extended period in vitro and exhibited a significant therapeutic effect in pharmacodynamic studies in vivo (HepG2 cells).	[133]
Nanoparticles	12-arm PEG-PCL	Docetaxel	Micelles		In vitro cytotoxicity (HeLa cells) study indicated a reduced cytotoxicity.	[113]

**Table 2:** The variation of thermal and crystallinity characteristics in accordance with the macromolecular architectures of PCL-based multi-arms.

Polymers	Type	Arms	T_C,PCL_	T_m_	T_g_	X_C_%	Molten State Morphology	Year	Ref.
-PEG_113_-b- PCL_102_ -PEG_113_-b-(HEMA-g-PCL_8_)_13_ -PEG_113_-b-(HEMA-g-PCL_5_)_26_	-Linear -Multiarms -Multiarms	1 13 26	27.6 −7 −17	58.6 53.1 45.1				2010	[165]
Star-shaped PCLs similar molecular weights (Mn ¼ 10 300)	Star shaped	1 2 3 4 5	– – – – –	57.5 55.1 54.9 53.1 52.9		61 57 55 53 50		2008	[156]
-PPEGA-b-(PCL-_5_)_2_ -PPEGA-b-(PCL-_10_)_2_	Multiarms	2 2	– –	32.5 45				2009	[166]
-PCL_40_-b-PS_59_ -(PCL_2_)_39_-b-(PS_2_)_61_	AB A_2_B_2_	1 4	27.7 17.2	55.7 54.5	102.5 103.1	32 17	Lamellar PCL cylinders.	2009	[85]
PEO_2k_-PCL_4.3k_ PEO_2k_-(PCL_2.1k_)_2_	AB AB_2_	Linear 2	34.4 30.5	53.5 47.4		47 40			[167]
-PCL -PBA -μ-(PBA)_2_(PCL)_4_	– – μ-A_2_B_4_	6	25 – −7.8	49.9 – 44.8	−56 22 −49.5_PCL_ 26.3_PBA_	66 – 26	– – Uniform nanofiber	2019	[168]
-4a-PCL_5k_-b-PDLA _1k_	4armed	−4 −4	23 80	50 140		66 13	Dense spherulites. No spherulites.	2014	[169]
-4a-PCL_5k_-b-PDLA _10k_									
LPCL_4.8k_ 3SPCL_3.7k_ 4SPCL_9.4k_ 6SPCL_13.9k_	Linear 3armed 4armed 6armed	1 3 4 6	– 55.3 54.5 52.4	58 – – –		65.3 57.8 56.3 54		2020	[162]
PCL_62K_ 3S-PCL_99K_ 4S-PCL_18K_	Linear Star Star	1 3 4	29 31 28	54 54 51	−70 −68 −66	47 47 51		2020	[170]
6s-PCL-b-PLA _(Mn=66.1k)_ 6s-PLA-b-PCL _(Mn=66.3K)_	6 _armed_ 6 _armed_	6 6		50.6_PCL_149_PLA_	−55_PCL_35.5_PLA_		Phase separation. No phase separation.	2022	[171]
				120_PLA_	−57_PCL_ 36_PLA_				
-lignin-g-PCL_1_ -lignin-g-PCL_29_ -lignin-g-PCL_37_	Grafted arms	1 29 37	21.5 16.7 7.1	49.7 47.9 58_liging_ 44.5_PCL_		43 34 22	High lamellar thickening. – Limited lamellar thickening.	2015	[172]

## Data Availability

Not applicable.
